# Distinct Genomic Features Characterize Two Clades of *Corynebacterium diphtheriae*: Proposal of *Corynebacterium diphtheriae* Subsp. *diphtheriae* Subsp. nov. and *Corynebacterium diphtheriae* Subsp. *lausannense* Subsp. nov.

**DOI:** 10.3389/fmicb.2018.01743

**Published:** 2018-08-17

**Authors:** Florian Tagini, Trestan Pillonel, Antony Croxatto, Claire Bertelli, Angela Koutsokera, Alban Lovis, Gilbert Greub

**Affiliations:** ^1^Institute of Microbiology, Department of Laboratory Medicine, Lausanne University Hospital, Lausanne University, Lausanne, Switzerland; ^2^Division of Pulmonology, Department of Medicine, Lausanne University Hospital, Lausanne, Switzerland; ^3^Division of Infectious Diseases, Department of Medicine, Lausanne University Hospital, Lausanne, Switzerland

**Keywords:** non-toxigenic diphtheria, comparative genomics, virulence, mobile genetic elements, CRISPR, pili, sortase

## Abstract

*Corynebacterium diphtheriae* is the etiological agent of diphtheria, a disease caused by the presence of the diphtheria toxin. However, an increasing number of records report non-toxigenic *C. diphtheriae* infections. Here, a *C. diphtheriae* strain was recovered from a patient with a past history of bronchiectasis who developed a severe tracheo-bronchitis with multiple whitish lesions of the distal trachea and the mainstem bronchi. Whole-genome sequencing (WGS), performed in parallel with PCR targeting the toxin gene and the Elek test, provided clinically relevant results in a short turnaround time, showing that the isolate was non-toxigenic. A comparative genomic analysis of the new strain (CHUV2995) with 56 other publicly available genomes of *C. diphtheriae* revealed that the strains CHUV2995, CCUG 5865 and CMCNS703 share a lower average nucleotide identity (ANI) (95.24 to 95.39%) with the *C. diphtheriae* NCTC 11397^T^ reference genome than all other *C. diphtheriae* genomes (>98.15%). Core genome phylogeny confirmed the presence of two monophyletic clades. Based on these findings, we propose here two new *C. diphtheriae* subspecies to replace the lineage denomination used in previous multilocus sequence typing studies: *C. diphtheriae* subsp. *lausannense* subsp. nov. (instead of lineage-2), regrouping strains CHUV2995, CCUG 5865, and CMCNS703, and *C. diphtheriae* subsp. *diphtheriae* subsp. nov, regrouping all other *C. diphtheriae* in the dataset (instead of lineage-1). Interestingly, members of subspecies *lausannense* displayed a larger genome size than subspecies *diphtheriae* and were enriched in COG categories related to transport and metabolism of lipids (I) and inorganic ion (P). Conversely, they lacked all genes involved in the synthesis of pili (SpaA-type, SpaD-type and SpaH-type), molybdenum cofactor and of the nitrate reductase. Finally, the CHUV2995 genome is particularly enriched in mobility genes and harbors several prophages. The genome encodes a type II-C CRISPR-Cas locus with 2 spacers that lacks *csn2* or *cas4*, which could hamper the acquisition of new spacers and render strain CHUV2995 more susceptible to bacteriophage infections and gene acquisition through various mechanisms of horizontal gene transfer.

## Introduction

Classical diphtheria is due to the production of a toxin during *C. diphtheriae* infections by strains lysogenized by a bacteriophage (corynephage) holding the toxin gene. Thanks to vaccination programs, the incidence of toxigenic diphtheria has dramatically decreased during the past century in industrialized countries (Kitchin, 2011). However, there has been a recent increase in non-toxigenic *C. diphtheriae* infections reported with various atypical clinical presentations including pharyngitis, respiratory tract infections, endocarditis, osteomyelitis, septic arthritis or cutaneous infections (Gubler et al., 1998; Romney et al., 2006; Hirata Jr et al., 2008; Edwards et al., 2011; Zasada, 2013; FitzGerald et al., 2015; Kolios et al., 2017; Okamoto et al., 2018). As a potential public health threat, toxigenic *C. diphtheriae* infections need to be detected. PCRs targeting the toxin encoding gene and the Elek test remain the standard to quickly characterize the toxigenic potential of an isolate (Efstratiou et al., 1994; Public Health England, 2015; De Zoysa et al., 2016). In Switzerland, clustered cases of cutaneous (toxigenic and non-toxigenic) diphtheria were recently reported in the migrant population and whole-genome sequencing was useful to rule out recent direct transmission of a clone (Meinel et al., 2016).

Besides the toxin, other virulence factors such as the three operons encoding for pili (SpaA cluster, SpaD cluster and SpaH cluster) and genes related to iron-uptake may play a role in *C. diphtheriae* infections (Trost et al., 2012). The regulation of virulence is mainly due to the Diphtheria toxin Repressor (DtxR), which binds the promoter and represses in an iron-dependant manner the transcription of the toxin gene as well as numerous genes involved in iron homeostasis (Schmitt and Holmes, 1991b; Lee et al., 1997; Schmitt et al., 1997). In low-iron conditions, such as in the human host, the repression of the DtxR is released, leading to the transcription of the toxin (Boyd et al., 1990; Schmitt and Holmes, 1991a,b).

*C. diphtheriae* was historically classified into four biovars—gravis, mitis, intermedius, and belfanti—based on biochemical phenotypic testing (Funke et al., 1997; Goodfellow et al., 2012). However, *C. diphtheriae* strains within a certain biovar can be genetically more distant than between biovars (Trost et al., 2012; Sangal et al., 2014). Thus, genomics does not support the use of biovars to reliably classify *C. diphtheriae* isolates (Sangal and Hoskisson, 2016). In addition, there is a lack of correlation between biovar determination and pathogenicity (Bolt et al., 2010). Multilocus sequence typing (MLST), based on the allelic determination of 7 house-keeping genes, has recently been used to separate two distinct lineages, called lineage-1 (comprising most strains) and lineage-2 (regrouping only biovar belfanti strains) (Bolt et al., 2010). A third lineage was described by Farfour et al. but currently only one strain is known to belong to this sequence-type (Farfour et al., 2013).

Confronted with a very particular clinical presentation and bronchoscopy findings in a patient, WGS was applied to a *C. diphtheriae* isolate, of strain CHUV2995, to exclude the presence of the diphtheria toxin in a clinically relevant turnaround time, in parallel to a specific PCR for the toxin gene and an Elek test. Then, a comparative genomic analysis was performed to investigate the particular genomic features of strain CHUV2995 as well as the presence of virulence factors. The biochemical phenotype was also characterized to better describe this *C. diphtheriae* strain.

## Materials and methods

### Bacterial strain and growth conditions

*C. diphtheriae* CHUV2995 was isolated from the bronchoalveolar lavage (BAL) of a patient hospitalized in Lausanne University Hospital and subsequently identified using a matrix-assisted laser desorption ionization time-of-flight mass spectrometry (MALDI-TOF MS) (Bruker, US). For Ion Torrent as well as for API Coryne (bioMérieux) identification tests (see below), bacteria were grown on blood agar plates at 37°C in a 5% CO_2_ humidified atmosphere for 24–48 h. For PacBio sequencing (see below), bacteria were grown in Todd-Hewith Broth (THB) at 37°C in ambient atmosphere for 48–72 h.

### DNA extraction and sequencing

Genome sequencing was done using two different technologies: PGM Ion Torrent (Life Technologies, Carlsbad, US) technology was used in order to exclude the presence of the toxin in a clinically relevant time frame. The genome was then re-sequenced using a PacBio RSII (Pacific Biosciences, Menlo Park, CA, US) to assemble the numerous repetitive regions of the genome that could not be properly assembled from short read data. Only the bioinformatics analyses performed on the PacBio data are detailed and reported here.

#### Ion torrent PGM sequencing

Genomic DNA extraction and purification were performed using the protocol for Gram-positive bacteria with the Wizard Genomic DNA Purification Kit (Promega, ref. A1120). Libraries were prepared using the Ion Xpress Plus Fragment Library Kit (Life Technologies, ref. 4471269) and the Ion Xpress Barcode Adapters 1–16 Kit (Life Technologies, ref. 4471250). Sequencing of 100 base pairs (bp) paired-reads was done using a PGM Ion Torrent (Life Technologies). Five independent runs were performed in order to achieve a sufficient theoretical coverage for the analysis.

#### Pacific biosciences RS II sequencing (pacific biosciences)

Hundred milliliters of culture in the exponential phase (THB medium) were used to obtain enough good quality DNA. Each culture was centrifuged for 2 min at 16,000 g and resuspended in 600 μl of a 4 mg/ml lysozyme solution diluted in EDTA 50 mM. Samples were incubated for 2 h and centrifuged 2 min at 16,000 g. The next purification steps were performed using the Wizard SV Genomic DNA Purification System (Promega, ref. A2361). DNA was finally eluted in 10 mM TRIS pH 8.0. Sequencing was performed on a Pacific Biosciences RS II sequencer using one SMRT cell of chemistry version P6-C4 (Pacific Biosciences, Menlo Park, CA, US). The 131,813 reads obtained presented a mean length of 10,577 bp.

### Assembly

*De novo* assembly of the PacBio sequences of CHUV2995 was carried out using the Hierarchical Genome Assembly Process (HGAP) workflow (PacBio DevNet; Pacific Biosciences, Menlo Park, CA, US), as available in SMRT Analysis v2.3.0. The assembly contained 3 contigs: 1 main circularized contig of 3,088,235 bp, 1 small circularized contig of 22,088 bp and 1 small linear contig of 29,039 bp. Pacbio reads were mapped on the assembly using Burrows-Wheeler Aligner (BWA-SW) v0.7.12 and SAMtools v1.2 (Li et al., 2009; Li and Durbin, 2010). Mapping quality and coverage were assessed using Qualimap v2.2. A coverage drop could be seen in the main contig between positions 2,903,566 and 2,960,750 as well as on the small contigs. Since BWA assigns randomly reads that can equally match different locations in a genome, coverage drop can be seen in wrongly duplicated regions in an assembly. Wrongly duplicated regions were identified using Genome Pair Rapid Dotter (GEPARD) (Krumsiek et al., 2007), and the sequence similarity was further confirmed using Mafft v7.187 (no SNPs could be seen between the duplicated sequences) (Katoh and Standley, 2013). Bases between position 2,931,515 and 2,959,386 of the main contig, as well as the two small contigs, were removed from the assembly. Following that, read mapping showed a uniform coverage across the final 3,060,363 bp chromosome.

### Genomes included in the analysis

All the *C. diphtheriae* strains indicated in Table S1, as well as *C. ulcerans* BR-AD22, were included for the core genome phylogeny and the subsequent comparative genomic analysis. The Average Nucleotide Identity (ANI) was calculated between all pairs of genomes.

### Annotation

The CHUV2995 genomic sequence, all the genomes from the University of Basel and the strain TH2031 were annotated using Prokka v1.11 (Seemann, 2014). For all the other genomes, annotation was already provided on RefSeq database (or Genbank if RefSeq annotation was not available). Protein domains were predicted using InterProScan v5.18-57.0 (Jones et al., 2014) and Pfam (Finn et al., 2014). A BLASTP search for every protein sequence was performed against the Clusters of Orthologous Groups database (Galperin et al., 2015), as available on the National Center for Biotechnology Information (NCBI) server (ftp://ftp.ncbi.nih.gov/pub/COG/COG2014/data/prot2003-2014.fa.gz). BLASTP algorithm v2.3.0+ was used with cut-offs of 10^−5^ for *e*-value, 20% for amino acid identity and 50% of query coverage (Altschul et al., 1997). In addition, KEGG Orthology (KO) numbers were assigned using GhostKOALA v2.0 (Kanehisa et al., 2016). DOOR 2.0 database was used to look for gene operons (Mao et al., 2014).

### Average nucleotide identity

The average nucleotide identity (ANI) was calculated using NUCmer v3.1, a tool of the MUMmer software (Kurtz et al., 2004). CHUV2995 strain and NCTC 11397^T^ were both used as a reference for the pairwise calculation. All the genomes of the strains described in Table S1, except the two references (CHUV2995 and NCTC11397^T^, respectively), were used as input to calculate the ANI.

### Prediction and comparison of groups of orthologs/paralogs

Orthofinder v1.1.4 was used to predict and to cluster orthologs and paralogs into so-called “orthogroups” (Emms and Kelly, 2015). In order to compare the presence/absence of orthogroups in the genomes of all *C. diphtheriae* and *C. ulcerans* strains, data was loaded and compared in MySQL 5.7.18 using homemade scripts that could query the tables of orthogroups, InterPro domains, Pfam motifs, COG and KO using MySQL syntax. Classical virulence factors, as previously described in (Allen and Schmitt, 2011; Trost et al., 2012; Sangal et al., 2015) were analyzed (Data Sheet 1).

### Core genome phylogeny

Core genome alignment was built in two steps: first, amino acid sequences of single-copy orthologous genes of all the *C. diphtheriae* and *C. ulcerans* strains were aligned using Mafft v7.187 (Katoh and Standley, 2013) and concatenated. Then, FastTree v2.1.8 (Price et al., 2010) was used to generate the core genome phylogeny (parameters: “–gamma –spr 4 –mlacc 2 –slownni”).

### Prophages, genomic islands and crisprs regions

PHASTER (Arndt et al., 2016) and IslandViewer 4 (Bertelli et al., 2017) were used to predict prophages and genomic islands, respectively. Genomic islands of CHUV2995 were also detected using a homemade script highlighting genomic regions larger than 4,500 bp that did not align with NUCmer in more than 80% of strains. In addition, these genomic regions were merged when less than 2,000 bp apart to tackle the issue of small repeated genomic regions. Finally, CRISPRfinder was used (Grissa et al., 2007) to identify Clustered Regularly Interspaced Short Palindromic Repeats (CRISPR).

### Multilocus sequence typing

Multilocus sequence typing was performed on all included strains using the mlst software (Seemann T, mlst, Github https://github.com/tseemann/mlst) based on the *C. diphtheriae* scheme as available from PubMLST (https://pubmlst.org) (Jolley and Maiden, 2010). To evaluate the worldwide distribution of lineage-2 (Bolt et al., 2010), the concatenated sequences of the seven housekeeping genes of each *C. diphtheriae* ST were retrieved from pubMLST (*n* = 541, May 2018). All sequences were aligned using mafft v7.187 and a phylogeny reconstructed with FastTree v2.1.8 (with parameters “–nt”) (Price et al., 2010; Katoh and Standley, 2013).

### Phenotypic testing

API Coryne (bioMérieux) identification test was used to test the metabolic features of the strain CHUV2995 according to the manufacturer's instructions.

## Results

### Clinical case report

A young adult patient originally from South-East Asia, in Switzerland for several years, was admitted to the Lausanne University Hospital for persistent painful cervical lymphadenopathies without fever or weight loss. He had a medical history of lymph node and pulmonary tuberculosis complicated by apical bronchiectasis, chronically colonized by *Pseudomonas aeruginosa*. The patient presented with a 2-week history of asthenia, odynodysphagia, purulent rhinorrhea, dry cough, hemoptoic sputa and reported four episodes of loss of consciousness.

Clinical examination was normal except for a modest pharyngeal hyperemia and a submandibular painful lymphadenopathy (2 × 2 cm). A few other smaller cervical lymphadenopathies were present.

**Figure 1:**
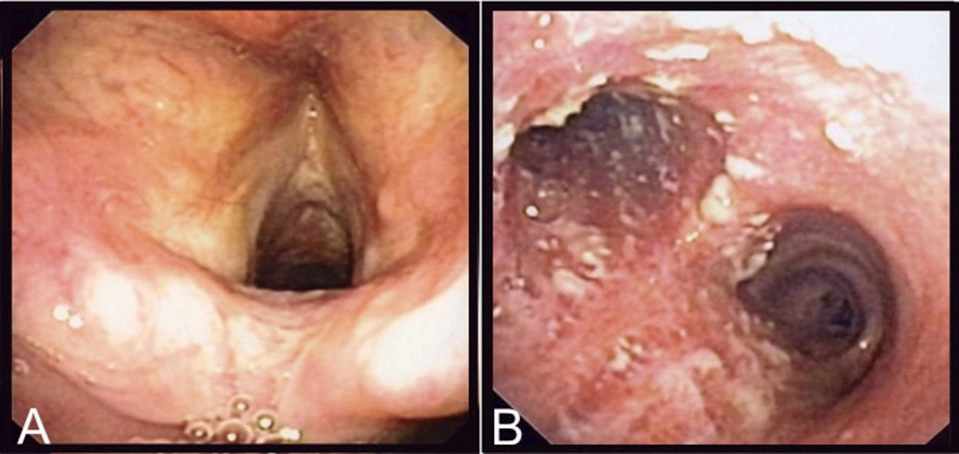
Endoscopic findings of the patient. On **(A)**, are shown the normal epiglottis and larynx of the patient and on **(B)**, the adherent whitish lesions of the distal trachea and both mainstem bronchi.

Laboratory analyses revealed a normal C-reactive protein (CRP) (5 mg/l) and normal complete blood count. Renal and hepatic functions were also normal. A chest X-ray was similar to the one performed 1 year before and a thoracic CT-scan confirmed the presence of right upper lobe bronchiectasis without any additional findings. A fine needle biopsy of the submandibular lymphadenopathy showed a non-specific inflammatory process, without granulomas; PCR and cultures for *Mycobacterium tuberculosis* and other mycobacteria were negative. Serologies for HIV, CMV, EBV, *Toxoplasma gondii, Bartonella* spp. were also negative. To exclude tuberculosis, a bronchoscopy was performed, that showed normal features up to the proximal trachea (Figure 1A). The carina and both mainstem bronchi had unusual multiple adherent whitish lesions (Figure 1B). The lobar and segmental bronchi exhibited no lesions. Histologic examination of bronchial biopsies revealed chronic inflammation (malpighian hyperkeratotic mucosa). The BAL showed alveolar lymphocytosis (65%). Gram staining of the BAL revealed more than 25 leucocytes per microscopic field and some Gram-positive bacilli. *C. diphtheriae* (10^3^ CFU/ml) was recovered after culture of the BAL sample and identified by MALDI-TOF (Croxatto et al., 2012) together with, as expected, colonizing *P. aeruginosa* (10^4^ CFU/ml).

The *C. diphtheriae* strain isolated, CHUV2995, was found to be susceptible to all antimicrobial agents tested (penicillin, amoxicillin, clindamycin, levofloxacin, ciprofloxacin, erythromycin and azithromycin). The patient was successfully treated with erythromycin for 14 days. Cardiac investigations did not reveal arrhythmia nor conduction disturbances, precluding the presence of cardiotoxicity. Given the unusual clinical presentation with chronic severe tracheobronchitis, the bacterial genome was sequenced for characterization and exclusion of the presence of the toxin gene for both clinical and epidemiological reasons (the patient came from a center for migrants). The genome analysis, specific PCR and Elek test all confirmed that the *C. diphtheriae* strain was non-toxigenic.

Here, the acute clinical manifestations of this patient were attributed to *C. diphtheriae* considering (1) the bronchoscopic findings, (2) the previously documented presence of *P. aeruginosa* indicating colonization rather than *de novo* infection, and (3) the clinical improvement after macrolide use (an antibiotic class lacking significant antipseudomonal properties).

### Strain CHUV2995 is part of a distinct *C. diphtheriae* clade with particular genomic features

**Figure 2:**
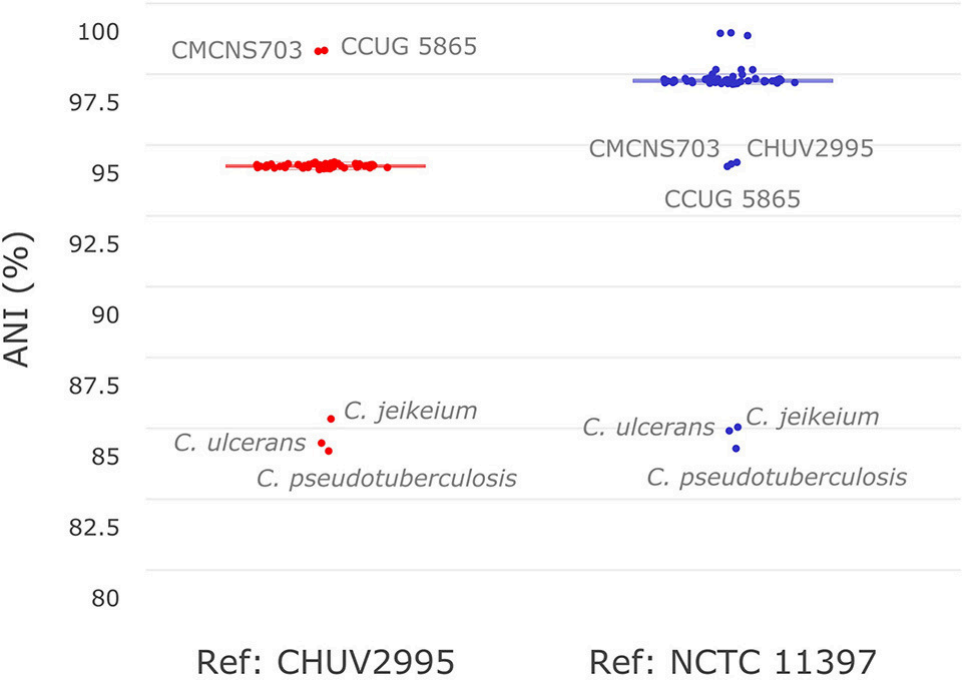
Average nucleotide identity. Pair-wise average nucleotide identities (ANI) with all *C. diphtheriae* strains as well as other closely related species of the same genus (*C. ulcerans, C. pseudotuberculosis*, and *C. jeikeium*) were calculated using *C. diphtheriae* strain CHUV2995 and *C. diphtheriae* NCTC 11397^T^ as reference. As shown here in this boxplot (very condensed around the median), all *C. diphtheriae* strains but CCUG 5865 and CMCNS703 share an ANI between 95 and 96% when compared to strain CHUV2995. Conversely, when using *C. diphtheriae* strain NCTC 11397^T^ as reference, the ANI shared with all *C. diphtheriae* is above 98%, except for CHUV2995, CCUG 5865 and CMCNS703. Therefore, two groups of strains were observed, one with CHUV2995, CCUG 5865, and CMCNS703 and the other with the rest of *C. diphtheriae* strains.

To investigate the similarity of *C. diphtheriae* strain CHUV2995 to other strains available in sequence databases, pairwise ANI calculations were performed with the references CHUV2995 and NCTC 11397^T^ genomes (median alignment coverage 79.89 and 82.44%, respectively). Strain CHUV2995 shared a median ANI of 95.25% (Figure 2) with all strains included in the analysis (Table S1), except CCUG 5865 and CMCNS703 that were more closely related (>99% ANI). By contrast, NCTC 11397^T^ shared more than 98.15% ANI with all *C. diphtheriae* strains except CHUV2995, CMCNS703, and CCUG 5865. Thus, CHUV2995, CCUG 5865, and CMCNS703 are closely related, and cluster separately from all other *C. diphtheriae* strains. Despite the relatively large differences in ANI between groups, 16S rRNA gene conservation between all *C. diphtheriae* strains was above 99% identity, suggesting that the new clade should be classified as part of the *C. diphtheriae* species (data not shown). Interestingly, CCUG 5865 was recently used by Grosse-Kock et al. (2017) as an outgroup for their core genome phylogeny because it belonged to lineage-2. The multilocus sequence types of CHUV 2995 and CMCNS703 also cluster with lineage-2 (Figure S1 and Data Sheet 3). Given the large genomic distances, we propose to rename lineage-2 as *C. diphtheriae* subsp. *lausannense* subsp. nov. (regrouping *C. diphtheriae* strains CHUV2995, CMCNS703, and CCUG 5865) and lineage-1 by *C. diphtheriae* subsp. *diphtheriae* subsp. nov. (regrouping the other genome sequenced *C. diphtheriae* strains analyzed).

**Figure 3:**
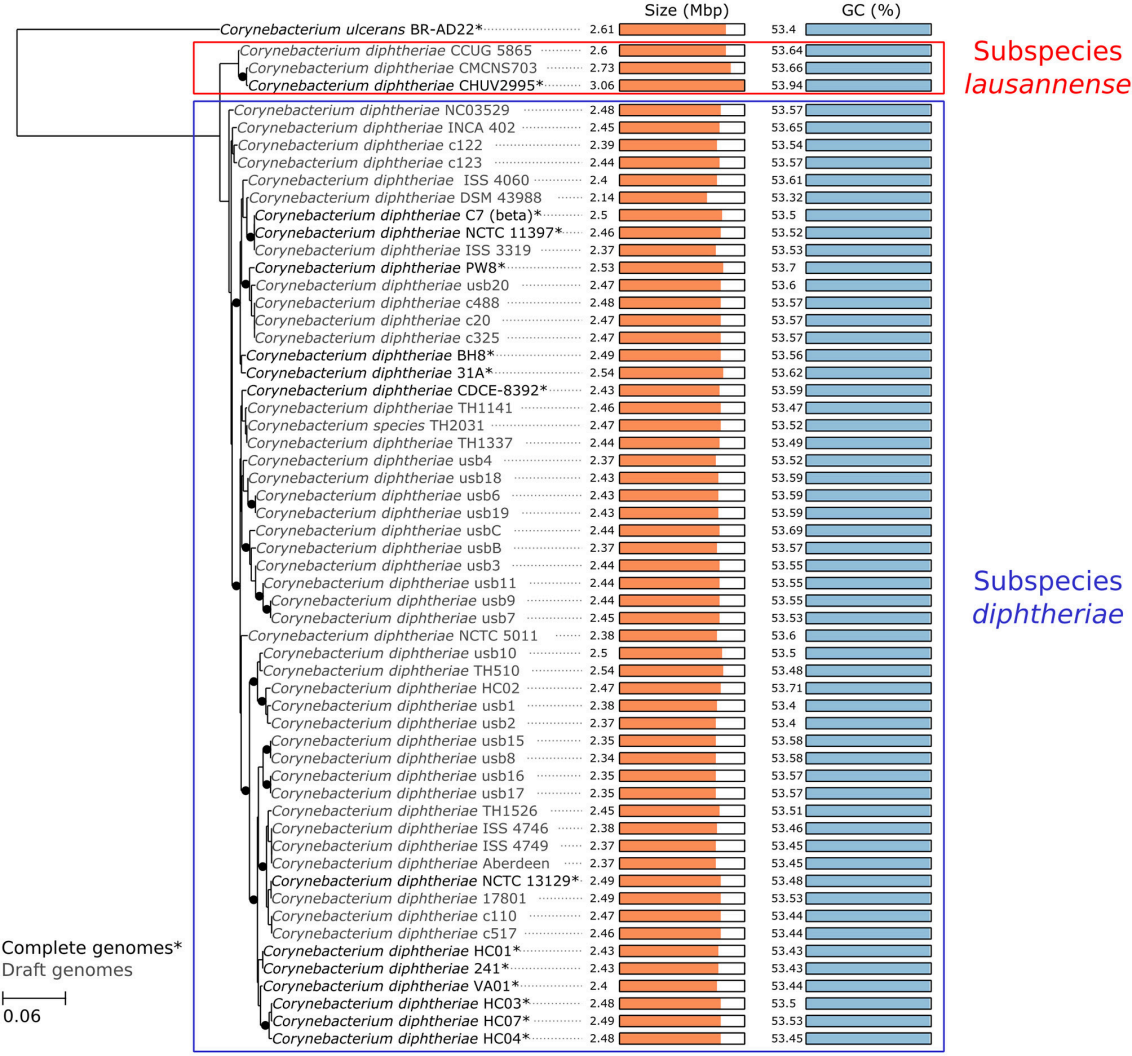
Core genome phylogeny. A maximum likelihood phylogenetic tree was reconstructed based on the concatenated alignment of single-copy orthologous genes belonging to the *C. diphtheriae* and *C. ulcerans* core genome. The scale bar represents the number of amino acid substitutions per site alongside the branches. Nodes supports are based on the Shimodaira-Hasegawa (SH) test. Black dots indicate when node values are below 1 (lines are shifted to the right to accommodate the presence of the dots, which should not be considered as phylogenetic distances). CHUV2995 clusters with two closely related isolates, defining the monophyletic subspecies *lausannense*, whereas the other strains cluster in subspecies *diphtheriae*. Interestingly, CHUV2995 displays the largest genome of the dataset.

To confirm the monophyly of the new subspecies, the amino acid translation of core genes common to all *C. diphtheriae* strains as well as *C. ulcerans*—used as an outgroup—were aligned (total length 323,259 amino acids) and used to reconstruct a maximum-likelihood tree. The phylogenetic tree is consistent with the ANI calculations and shows that strain CHUV2995 forms a monophyletic clade with CCUG 5865 and CMCNS703, whereas members of subspecies *diphtheriae* form another distinct clade (Figure 3). Interestingly, subspecies *lausannense* genomes are significantly larger (CHUV2995, 3.06 Mb; CCUG 5865, 2.6 Mb; CMCNS703, 2.73 Mb) than subspecies *diphtheriae* genomes (*p* = 0.0040, Wilcoxon rank sum test was used because data distributions were asymetrical in both groups). Two-sample *t*-test comparing GC content between the two clades was not significant (*p* = 0.1555). However, the genome of strain CHUV2995 displayed the highest GC content (53.94%), whereas the mean GC content of all other *C. diphtheriae* strains in the dataset is 53.54 ± 0.16%.

### Virulence factors of subspecies *lausannense*

**Figure 4:**
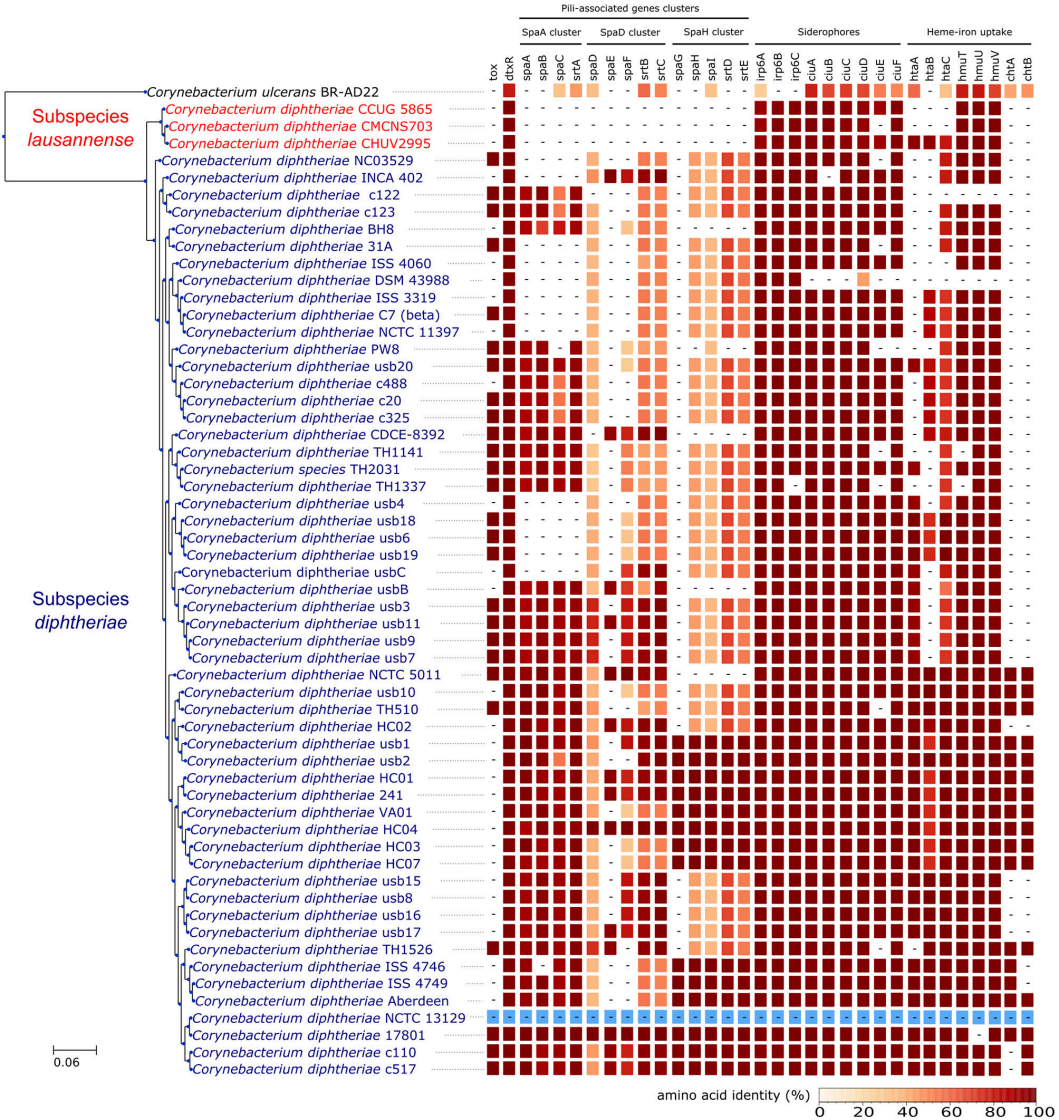
Virulence factors. CHUV2995, CUG 5865, and CMCNS703 (Subspecies *lausannense*) present no gene encoding for—or associated to—the classical *C. diphtheriae* pili operons. Interestingly, even *C. ulcerans* encodes homologs of these genes. CHUV2995 genome encodes more genes related to heme-iron uptake than the other strains of subspecies *lausannense*. NCTC13129 sequences were used as a reference to compute the amino acid identity (blue).

Orthologs of known virulence factors were identified in the genomes of the dataset (Figure 4 and Data Sheet 1). Interestingly, the strains belonging to subspecies *lausannense* had no orthologs of the pili-associated genes (located on gene operons encoding for SpaA-type, SpaD-type and SpaH-type pili) based on OrthoFinder analysis and additional tBLASTN searches, which is consistent with a previous study (Grosse-Kock et al., 2017). No InterPro domain or Pfam motifs related to these pili-associated genes were found in subspecies *lausannense* genomes (except for domains that are not pili-specific), thus further supporting the lack of pilus (Data Sheet 1). Similarly, no pili-associated sortases (*srtA, srtB, srtC, srtD*, and *srtE*) could be identified in subspecies *lausannense* (Data Sheet 1). Only class E sortases (such as *srtF*) which act as housekeeping sortases in *C. diphtheriae* (Swaminathan et al., 2007; Spirig et al., 2011) were identified in subspecies *lausannense*. CHUV2995 has a supplementary sortase-family protein (CHUV2995_00246) that shared high amino acid identity (96%) with other pili-associated sortases (class C) but had a small size of 114 amino acids and only one transmembrane domain as compared to a size of ~300 amino acids and two transmembrane domains for classical pili-associated sortases of *C. diphtheriae*. Unlike pili-associated sortases, this gene was not located in a gene cluster related to pili and its function remains uncertain.

Regarding iron uptake, the CHUV2995 genome encodes orthologs of *htaA, htaB*, and *htaC* (Figure 4 and Data Sheet 1), genes that are involved in heme-associated acquisition of iron (Allen and Schmitt, 2009). These genes shared a high amino acid sequence identity to homologs in strain NCTC 13129, ranging from 80.62% (*htaC*) to 98.39% (*hmuT*). Orthologs of those genes were not identified in the two other subspecies *lausannense* strains.

The three genomes of subspecies *lausannense* did not harbor the complete sequence of the toxin gene based on orthologs prediction and on the absence of complete Pfam motifs and InterPro domains corresponding to the toxin (Data Sheet 1). However, two overlapping fragments of the R domain of the diphtheria toxin (101 and 38 amino acids, respectively, and overlapping on 34 amino acids), were detected on two small contigs of the strain CMCNS703 genome. Sequence identity with the toxin of NCTC 1329 using BLASTP was 100 and 87.17% for the large and the small fragments, respectively. The presence of a complete but not well-assembled toxin in CMCNS703 genome is unlikely since we did not find another fragment of the toxin in the assembly. Alternatively, remnants of the toxin may be present if the gene is undergoing pseudogenisation and progressive gene loss. Finally, the NCTC 13129 toxin region was compared to the corresponding CHUV2995 region, which lacks the toxin but harbors sequences of another prophage inserted at the same genomic position (Figure S2).

The translated sequence of the *dtxR* gene was completely identical in the three strains of subspecies *lausannense* and shared 97.79% amino-acid identity with those of subspecies *diphtheriae*. It was also identical to that described by Dinu et al. (2014), who reported this allele in both toxigenic and non-toxigenic, toxin gene-bearing isolates, suggesting that this allele could efficiently regulate the toxin gene, if the strain gets lysogenized.

**Figure 5:**
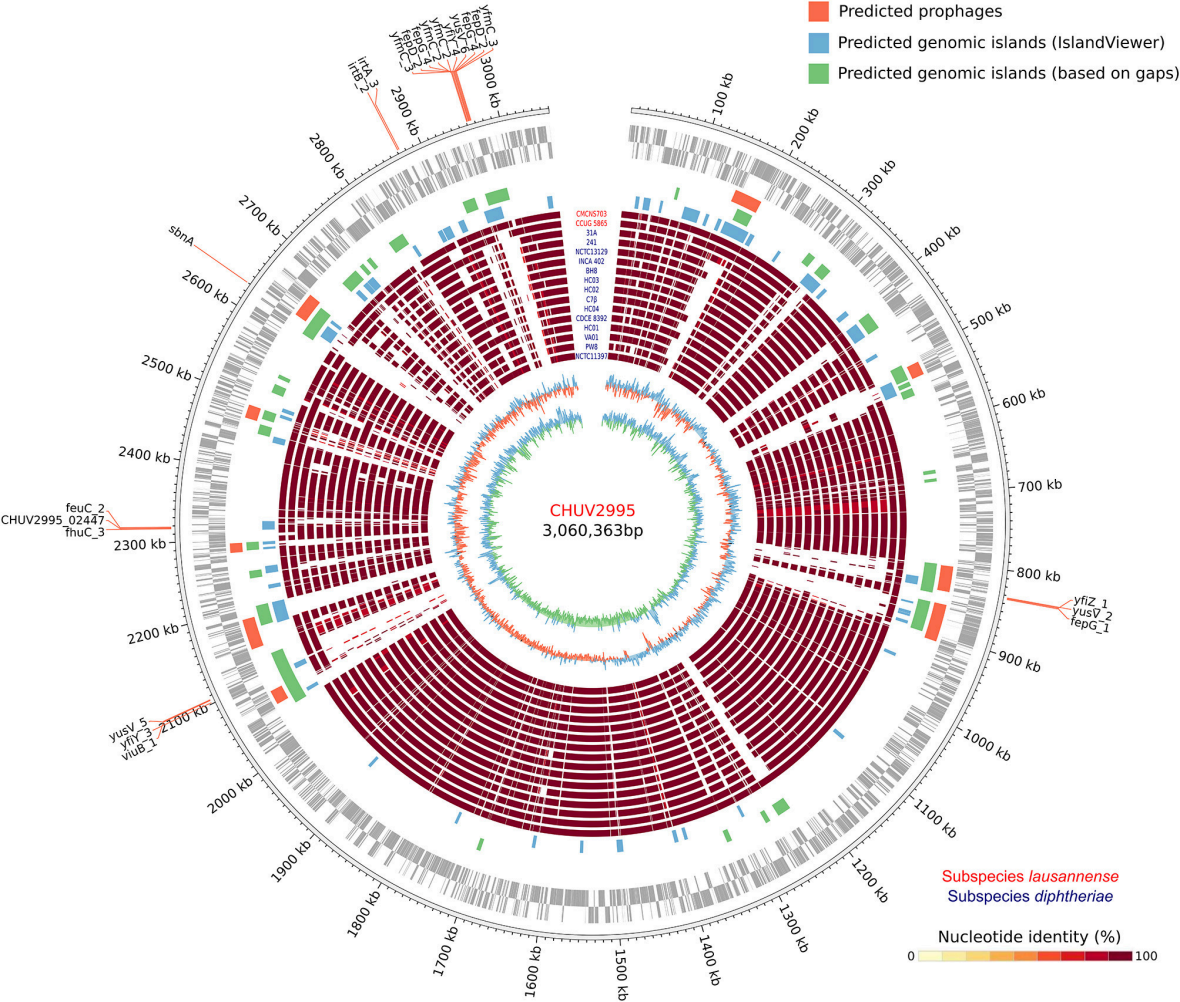
Circular genome representation. Circles from inside to outside represent the GC content, GC skew, 15 complete genome sequences of *C. diphtheriae* strains as well as the two genome sequences of subspecies *lausannense* (red), genomic islands (GIs) predicted using IslandViewer 4 (blue), a homemade script (green; regions of ≥4.5 Kb not aligning with NUCmer in more than 80% of the strains), PHASTER (orange; prophages), open reading frames (ORFs) on the lagging strand and on the leading strand (gray) and chromosomal genomic positions. Genes probably involved in the iron uptake (based on automatic annotation) present on the GIs were annotated, thus delineating putative pathogenicity islands.

The large genome size could be explained by the large number of genomic islands identified and in particular those formed by prophages in the genome of CHUV2995 (Figure 5). Some of them were conserved in the genomes of the other strains of subspecies *lausannense*. Several genes encoding for putative siderophores or putative heme-iron uptake systems were detected on the predicted genomic islands. These genes could thus indicate potential pathogenicity islands.

### Differences in metabolic capabilities

**Figure 6:**
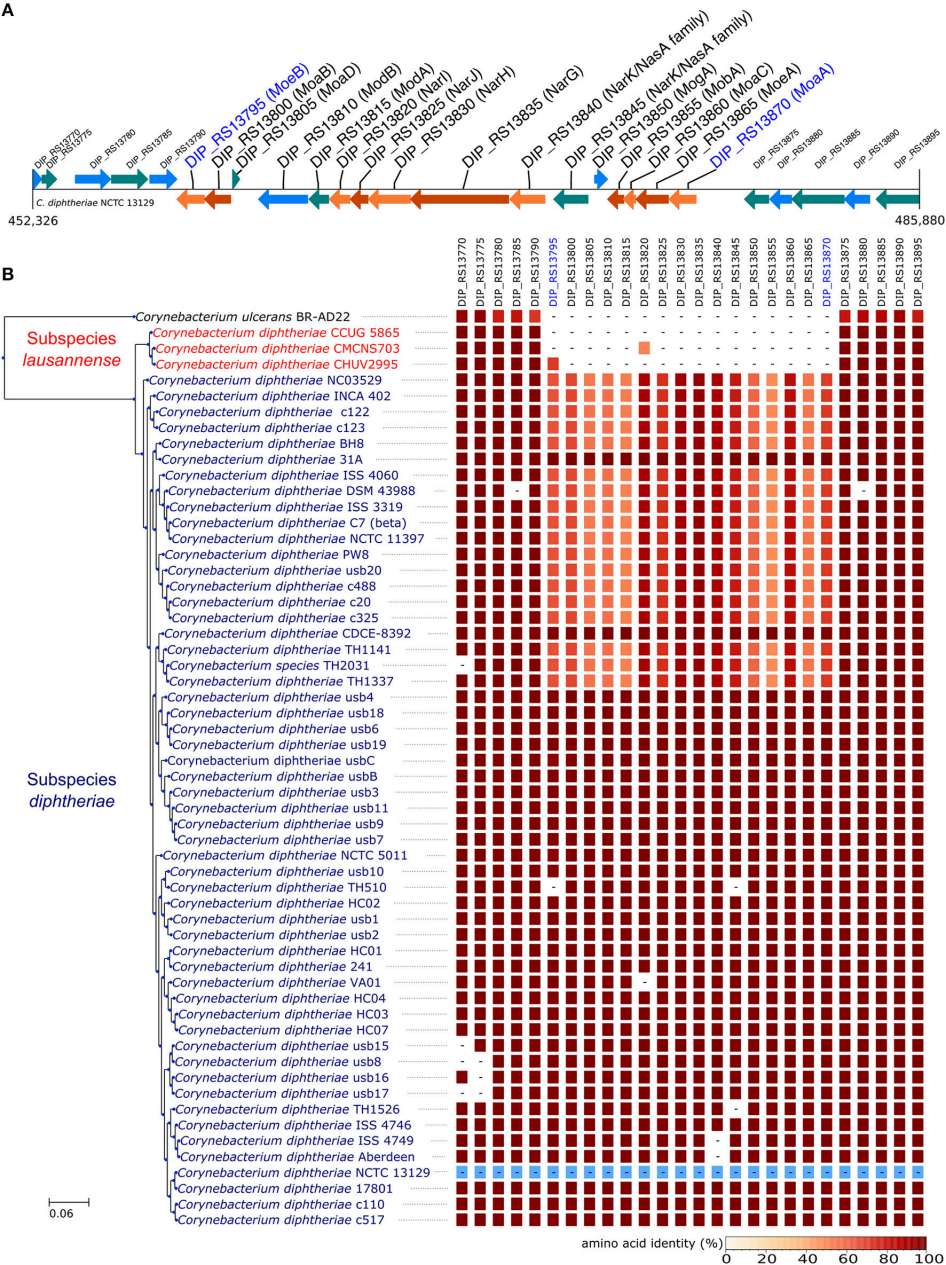
Missing nitrate reductase and molybdenum cofactor biosynthesis genes in subspecies *lausannense*. **(A)** The genomic region of the NCTC13129 genome involved in nitrate reduction and molybdenum cofactor biosynthesis is shown. Sequences in brown and orange indicate genes functioning in operon (predicted using DOOR 2.0), reference locus tags are indicated above the coding sequences and the predicted products are written between parenthesis, locus tags in blue indicate the border of the genomic region (corresponding to the locus tags in blue of **B**); **(B)** NCTC13129 genomic region (in light blue) was used as a reference to look for orthologs of genes of the genomic region of interest. Each box represents the presence/absence of an ortholog in each genome of the dataset. The color gradient indicates the amino-acid identity as compared to the reference sequence. The putative ortholog of the gene encoding for the nitrate reductase subunit beta in CMCNS703 is either due to a wrong clustering of orthologous proteins or to an assembly artifact (it is located on a small contig). The sequence alignment covers less than 50% of the reference sequence DIP_RS13820 (locus tag of strain NCTC13129) and could be a remnant of the nitrate reductase subunit beta. Similarly, the presence of an ortholog to DIP_RS13795, encoding for MoeB, in CHUV2995 is questionable: amino acid identity was 72.49% but with a coverage of less than 60% of the reference sequence. Therefore, we concluded that the genomic region encoding for the nitrate reducase and the molybdenum cofactor biosynthesis was absent in subspecies *lausannense*.

In order to identify differences in metabolic capabilities between subspecies *lausannense* and subspecies *diphtheriae*, we compared KO entries found only in subspecies *lausannense* or subspecies *diphtheriae*. Fourteen KO entries were specific to subspecies *lausannense* (Table 1) and among them, four were related to ABC transporters (3 zinc and 1 cobalt/nickel transport system). The other KO entries were related to amino acid, sugar or fat metabolism. Conversely, 9 KO entries were absent from subspecies *lausannense* and present in subspecies *diphtheriae* (Table 2). These KO were related to either nitrate reduction or molybdenum cofactor biosynthesis. Interestingly, all genes annotated with these KO entries were encoded in the same genomic region (Figure 6). More specifically, genes encoding for nitrate reductase subunits alpha (*narG*), beta (*narH*), gamma (*narI*) and delta (*narJ*) encoded in an operon together with *narK* (a major facilitator superfamily transporter) and *modA-B* (a molybdenum ABC transporter) were absent from subspecies *lausannense*. In addition, the adjacent operons implicated in molybdenum import and cofactor biosynthesis *moeBR-moaE(moaB)* and *mobA-moaC-moeA-moaA* were also absent from subspecies *lausannense* (Figure 6), similarly to previous observations in *C. pseudotuberculosis* (Viana et al., 2017).

**Table 1:** KO entries specific to subspecies *lausannense* and absent from subspecies *diphtheriae*.

**KO**	**Name(s)**	**Description**	**Enzyme entry (EC)**	**KEGG pathways**
K00059	*fabG*	3-oxoacyl-[acyl-carrier protein] reductase	1.1.1.100	Fatty acid biosynthesis Biotin metabolism Biosynthesis of unsaturated fatty acids Fatty acid metabolism
K02006	*cbiO*	Cobalt/nickel transport system ATP-binding protein	–	ABC transporters
				
K02182	*caiC*	Crotonobetaine/carnitine-CoA ligase	6.2.1.-	–
K02777	PTS-Glc-EIIA, *crr*	PTS system, sugar-specific IIA component	2.7.1.-	Glycolysis/Gluconeogenesis Starch and sucrose metabolism Amino sugar and nucleotide sugar metabolism Phosphotransferase system (PTS)
K02804	PTS-Nag-EIIC, *nagE*	PTS system, N-acetylglucosamine-specific IIC component	–	Amino sugar and nucleotide sugar metabolism Phosphotransferase system (PTS)
K03327	TC.MATE, SLC47A, *norM, mdtK, dinF*	Multidrug resistance protein, MATE family	–	–
K03343	*puo*	Putrescine oxidase	1.4.3.10	Arginine and proline metabolism
K07045	K07045	Uncharacterized protein	–	–
K07250	*gabT*	4-aminobutyrate aminotransferase / (S)-3-amino-2-methylpropionate transaminase	2.6.1.19 2.6.1.22	Alanine, aspartate and glutamate metabolism Valine, leucine and isoleucine degradation Beta-Alanine metabolism Propanoate metabolism Butanoate metabolism
K09684	*pucR*	Purine catabolism regulatory protein	–	–
K09815	*znuA*	Zinc transport system substrate-binding protein	–	ABC transporters
K09816	*znuB*	Zinc transport system permease protein	–	ABC transporters
K09817	*znuC*	Zinc transport system ATP-binding protein	3.6.3.-	ABC transporters
K15866	*paaG*	2-(1,2-epoxy-1,2-dihydrophenyl)acetyl-CoA isomerase	5.3.3.18	Phenylalanine metabolism

**Table 2:** KO entries present in subspecies *diphtheriae* and absent from subspecies *lausannense*.

**KO**	**Name(s)**	**Description**	**Enzyme entry (ECs)**	**KEGG pathways**
K00370	*narG, narZ, nxrA*	Nitrate reductase, alpha subunit	1.7.5.1 1.7.99.4	Nitrogen metabolism Two-component system
K00371	*narH, narY, nxrB*	Nitrate reductase, beta subunit	1.7.5.1 1.7.99.4	Nitrogen metabolism Two-component system
K00373	*narJ, narW*	Nitrate reductase delta subunit	–	Nitrogen metabolism Two-component system
K02018	*modB*	Molybdate transport system permease protein	–	ABC transporters
K02020	*modA*	Molybdate transport system substrate-binding protein	–	ABC transporters
K02575	NRT, *narK, nrtP, nasA*	MFS transporter, NNP family, nitrate/nitrite transporter	–	Nitrogen metabolism
K03636	*moaD*	Molybdopterin synthase sulfur carrier subunit	–	Folate biosynthesis Sulfur relay system
K03637	*moaC*	Cyclic pyranopterin phosphate synthase	4.1.99.18	Folate biosynthesis Sulfur relay system
K03639	MOCS1, *moaA*	Cyclic pyranopterin phosphate synthase	4.1.99.18	Folate biosynthesis Sulfur relay system

### CHUV2995 is enriched in many COG categories

**Figure 7:**
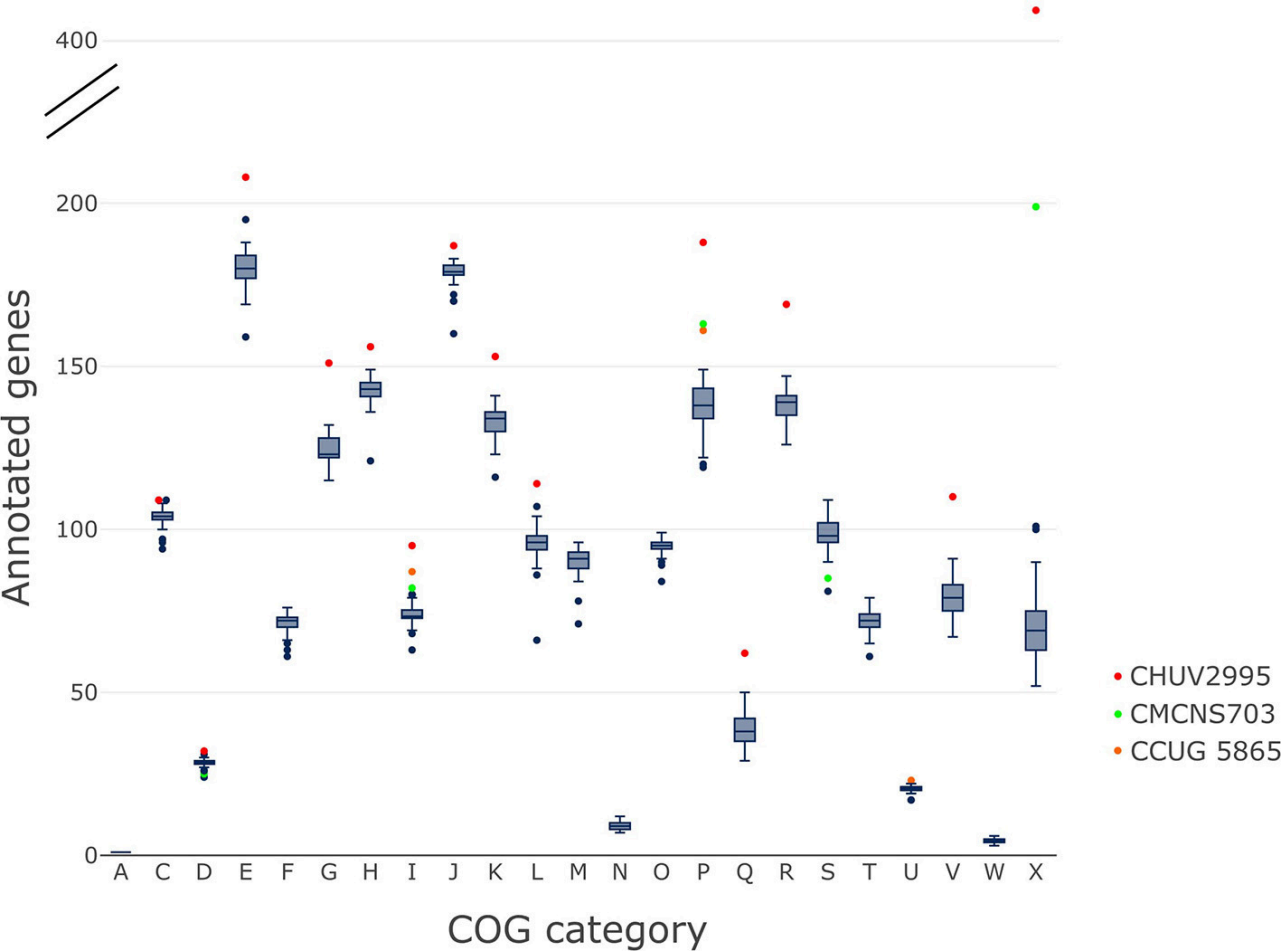
COG categories. The distribution of the number of genes per genome assigned to each COG category is shown on these boxplots.We indicated in red, CHUV2995; in green, CMCNS703 and in orange, CCUG 5865 when they are outliers. CHUV2995 was enriched in many COG categories and overall, subspecies *lausannense* was enriched in COG categories I and P. COG categories: A, Processing and modification; C, Energy production and conversion; D, Cell cycle control; cell division, chromosome partitioning; E, Amino acid transport and metabolism; F, Nucleotide transport and metabolism; G, Carbohydrate transport and metabolism; H, Coenzyme transport and metabolism; I, Lipid transport and metabolism; J, Translation, ribosomal structure and biogenesis; K, Transcription; L, Replication, recombination and repair; M, Cell wall/membrane/envelope biogenesis; N, Cell motility; O, Posttranslational modification, protein turnover, chaperones; P, Inorganic ion transport and metabolism; Q, Secondary metabolites biosynthesis, transport and catabolism; R, General function prediction only; S, Function unknown; T, Signal transduction mechanisms; U, Intracellular trafficking, secretion, and vesicular transport; V, Defense mechanisms; W, Extracellular structures; X, Mobilome: prophages, transposons.

The gene content of CHUV2995 was enriched (defined as being above the 90th percentile of the distribution) in many COG categories: E, Amino acid transport and metabolism, G, Carbohydrate transport and metabolism; H, Coenzyme transport and metabolism; I, Lipid transport and metabolism; J, Translation, ribosomal structure and biogenesis; K, Transcription; L, Replication, recombination and repair, P, Inorganic ion transport and metabolism; R, General function prediction only; V, Defense mechanisms and X, Mobilome: prophages, transposons (Figure 7). Surprisingly, the number of mobility genes (category X) is 8 fold higher than the median of all genomes and far above that of the 15 complete genome sequences included in the dataset. Indeed, repeated elements, such as mobility genes, can be underestimated in draft genomes due to the difficulty in assembling repeats. Categories C and D, related to energy production/conversion and cell cycle/division, respectively, are also slightly enriched in the CHUV2995 genome. Overall, subspecies *lausannense* is only enriched in categories related to lipid transport and metabolism (I) as well as to inorganic ion transport and metabolism (P) (Figure 7 and Figure S3). No more enrichment in most COG categories genes could be observed when subtracting COGs identified in CHUV2995 genomic islands (Figure S4 and Data Sheet 2), showing the large influence of additional genetic material acquired by CHUV2995 (Figure S5). Only categories G and I (carbohydrate and lipid metabolim) remained slightly enriched.

Given the enrichment of CHUV2995 in genes related to gene mobility and bacteriophages, it was also interesting to assess the presence of CRISPR-*Cas* systems in subspecies *lausannense* genomes, which could be involved in bacterial immunity against bacteriophages. A type II-C CRISPR-*Cas* system was identified in the CHUV2995 genome and included one single CRISPR region with only two spacers. The region is flanked by genes coding for a hydroxy/phosphor-methyl pyrimidine kinase and a hypothetical protein on one side, and an integrase core domain protein (a mobility gene) on the other side. Strain CCUG 5865 displayed two large CRISPR-Cas systems, one type I-E-a (19 spacers) and one type I-E-b system (also 19 spacers), whereas CMCNS703 only had 1 type I-E-a (3 spacers, and possibly more because the region is located at the border of a contig) (Figure S6).

### Phenotypic testing

The API *Corynebacterium* revealed a profile compatible with *C. diphtheriae* biovar mitis or belfanti. However, the alpha-glucosidase test was negative (Table S2), which is rather unusual because it was indicated as positive in 96% of strains of *C. diphtheriae* biovar mitis and belfanti in the table of identification provided by the manufacturer. Testing more strains belonging to subspecies *lausannense* would further indicate if the negativity of the alpha-glucosidase activity is systematic. Biovar belfanti strains are usually negative for the nitrate reductase activity despite the presence of the nitrate reductase operon. CHUV2995 was also negative for the nitrate reductase activity (Table S2), which could surprisingly be explained by the lack of the nitrate reductase operon (Figure 6), thus providing a genomic basis for the phenotypic results in this case.

### Subspecies *lausannense* has a worldwide distribution and is mostly isolated from the respiratory tract

Out of the 541 ST types available, 78 ST types can be identified as members of lineage-2 (the new subspecies *lausannense)* (Figure S7 and Data Sheet 3). By looking at the metadata of the 76 corresponding isolates (some ST types did not have corresponding isolates), 69 were recovered in Europe, 2 in Mayotte island, 3 in Algeria, 1 in Russia and 1 in Bangladesh. Interestingly, 27 isolates had a documented isolation site. The two most common isolation sites were upper (*n* = 10) and lower (*n* = 7) respiratory tract samples. Skin and wounds (*n* = 4) were the third most common isolation sites (Data Sheet 3).

## Discussion

In this study, we described the isolate CHUV2995 recovered from a patient with tracheobronchitis and propose to classify it in a new subspecies named *Corynebacterium diphtheriae* subsp. *lausannense* subsp. nov. For consistency, we propose the name *C. diphtheriae* subsp. *diphtheriae* subsp. nov. to regroup the clade containing most of *C. diphtheriae* isolates. Subspecies *diphtheriae* and *lausannense* correspond to lineage-1 and 2, respectively (Bolt et al., 2010; Grosse-Kock et al., 2017). Since the term “lineage” was also used for other ST types that are part of lineage-1 (du Plessis et al., 2017), we think that the use of subspecies could reduce confusion in the literature and help the reader to recognize clades showing these large genomic differences. An ANI cutoff of 95-96% has been proposed to distinguish between two bacterial species (Kim et al., 2014) and ANI values between subspecies *lausannense* and subspecies *diphtheriae* fall exactly within this range (Figure 2). However, the 99% nucleotide similarity of 16S rRNA shared between all *C. diphtheriae* isolates suggests the occurrence of a new subspecies rather than a new species. Indeed, accepted cutoffs for 16S rRNA nucleotide identity between strains of the same species range from 98.2 to 99% (Meier-Kolthoff et al., 2013; Kim et al., 2014). Lastly, core genome phylogeny confirmed the monophyly of the two subspecies.

The two other strains of subspecies *lausannense*, CCUG 5865 and CMCNS703, were isolated from nasal swabs in the United Kingdom and in India, respectively. Isolates belonging to lineage-2 were previously documented on 4 continents, showing a worldwide distribution of subspecies *lausannense* (Bolt et al., 2010; Farfour et al., 2013) (Data Sheet 3). Interestingly, several studies reported only lineage-1 (subspecies *diphtheriae*) isolates, suggesting that lineage-2 is less frequently encountered (Zasada, 2013; du Plessis et al., 2017; Grosse-Kock et al., 2017), as does the observation that ST affiliated to subspecies *lausannense* represent <15% of known ST.

Although all three subspecies *lausannense* strains studied encode *hmuTUV* genes involved the synthesis of the hemin ABC-transporter, CHUV2995 additionally encoded *htaA, htaB*, and *htaC*, associated to the binding and uptake of hemin (Allen and Schmitt, 2009). This suggests that CHUV2995 strain exhibits more pathogenic capabilities than the other subspecies *lausannense* strains.

Interestingly, CHUV2995 (together with the other strains of subspecies *lausannense*) presented no gene encoding for—or associated with—the classical *C. diphtheriae* pili operons despite the fact that the bronchoscopy showed adherent whitish lesions suggesting increased adhesive capabilities. However, the patient suffered from bronchiectasis, which is associated with retention of pulmonary secretions and could have promoted the adhesion of a strain with lower adhesive capabilities. Overall, subspecies *lausannense* might be less virulent than subspecies *diphtheriae* due to the lack of pili-associated (Broadway et al., 2013) as well as the nitrate reductase encoding genes, which is also known to promote virulence in other bacteria (Vázquez-Torres and Bäumler, 2016).

The genome of CHUV2995 was surprisingly highly enriched in most of the COG categories, suggesting that it has additional metabolic capabilities. All strains of subspecies *lausannense* had genes with KEGG Orthology functions that were not present in subspecies *diphtheriae*: namely functions involved in zinc and cobalt/nickel transport system or related to amino acid, sugar or lipid metabolism. In addition, COG category X, related to gene mobility and horizontal gene transfer, was notably enriched in CHUV2995, in line with the elevated number of predicted prophages and genomic islands (Figure 5). Interestingly, CHUV2995 harbored very few CRISPR spacers and encoded a type II-C Cas system lacking *cas4* or *csn2* (as discussed by Sangal et al., 2013; Jackson et al., 2017; Mir et al., 2018), both involved in the acquisition of new spacers in type II-A and II-B CRISPR-Cas systems. It remains unknown whether spacers may be acquired by II-C CRISPR-Cas systems on their own (Sangal et al., 2013; Jackson et al., 2017). The individual CRISPR-Cas systems identified in genomes of subspecies *lausannense* were previously described in *C. diphtheriae* by Sangal et al. (2013), but the concomitance of two type I-E (a and b type) CRISPR-Cas systems, as seen in CCUG 5865, was never reported before (Sangal et al., 2013; Hong et al., 2017).

Interestingly, subspecies *lausannense* specifically lacks genes encoding the nitrate reductase as well as genes involved in molybdenum cofactor biosynthesis. This genomic region was also found to be present in biovar equi and absent in biovar ovis of *C. pseudotuberculosis* and explains a positive nitrate reductase test for biovar equi (Viana et al., 2017). There is currently no genomic basis for the biovar classification of *C. diphtheriae* (Sangal and Hoskisson, 2016). Indeed, some strains, such as INCA 402, are classified as biovar belfanti, which are nitrate reducase negative although they contain the genomic region encoding the nitrate reductase enzymes. This suggests that nitrate reductase activity can depend on a difference at the transcriptional level for subspecies *diphtheriae* isolates. For subspecies *lausannense*, the lack of nitrate reductase activity can be explained by the absence of the genomic region involved in the synthesis of the nitrate reductase. Concerning the rest of the phenotypic findings, the API Coryne revealed CHUV2995 to be unusually negative for the alpha-glucosidase activity, which is rare for biovar belfanti (96% positive). However, no genomic explanation could be found since a gene encoding for alpha-glucosidase is present in CHUV2995 genome but may not have been expressed in the culture conditions tested. Practically, our data demonstrate that it is impossible to differentiate between the subspecies based on phenotypic data.

Since most analyses are based on the prediction of groups of orthologous proteins using OrthoFinder, the accuracy of protein clustering might have impacted our ability to identify orthologs in the two clades. Indeed, closely related genes of subspecies *lausannense* could have been clustered into a single group whereas the gene of interest of the reference strain could have clustered into a different group. This issue was tackled by performing searches for Pfam motifs and InterPro domains as well as BLAST searches of the protein of interest, which did not allow us to identify split groups of orthologs for the proteins of interest.

Our laboratory is not a reference center for *C. diphtheriae* and PCR for the toxin or Elek test is not routinely implemented in our diagnostic laboratories. Therefore, rapid microbial genomics provided information on the absence of the toxin in a short time frame, impacting on patient care and to prevent unnecessary isolation. It was particularly important to exclude the presence of the toxin gene given the fact that the patient was living in close proximity with many individuals with possible low vaccination rates, which would increase the risk of transmission. Currently, genomics represents an interesting alternative method to answer requests from clinicians, for instance when other simple tests are not available. Using genomics, the global gene content of an isolate—in this case virulence factors—can be quickly assessed, which has also proven useful in selected cases for other bacterial species (Tagini and Greub, 2017). In addition, whole genome sequencing prevents false negative results that can occur due to mutations in PCR target genes (Jaton et al., 2010). Overall, whole-genome sequencing provided interesting insights into *C. diphtheriae* strain diversity, unraveling large genomic differences between the two subspecies, and enabled us to provide the clinicians with meaningful clinical results in a short turnaround time.

## Description of *corynebacterium diphtheriae* subsp. *lausannense* subsp. nov.

*Corynebacterium diphtheriae* subsp. *lausannense* (lau.san.nen'se. N.L. neut. adj. *lausannense*, of Lausanne, a city in Switzerland, where the strain was isolated from a patient of the local University Hospital).

This subspecies was previously identified as lineage-2 in multilocus sequence typing (MLST) studies (Bolt et al., 2010; Farfour et al., 2013). Members of subspecies *lausannense* share an average nucleotide identity ranging from 95.24 to 95.39% with subspecies *diphtheriae*. Subspecies *lausannense* regroups only biovar belfanti strains. The type strain is CHUV2995^T^ = CCUG 72509^T^ = DSMZ 107520^T^, and its complete genome sequence can be found under the bioproject accession number PRJEB24256.

## Description of *corynebacterium diphtheriae* subsp. *diphtheriae* subsp. nov.

*Corynebacterium diphtheriae* subsp. *diphtheriae* (diph.the'ri.ae. Gr. fem. n. *diphthera*, piece of leather; N.L. fem. n. *diphtheria*, a disease in which leathery membranes form in the throat; N.L. gen. n. *diphtheriae*, of diphtheria).

*Corynebacterium diphtheriae* subsp. *diphtheriae* corresponds to lineage-1 in multilocus sequence typing studies (Bolt et al., 2010; Farfour et al., 2013) and was historically described as *Corynebacterium diphtheriae* by Kruse (1886) and Lehmann and Neumann (1896). The type strain is NCTC 11397^T^ = ATCC 27010^T^ = CIP 100721^T^ = DSM 44123^T^.

## Ethics statement

For single case report, we are exempted from ethical committee approval based on the rules from our local ethical committee.

## Author contributions

FT designed the study, performed the analyses and wrote the manuscript. TP contributed to part of the analysis. AC performed the initial WGS investigation in a short turnaround time, the phenotypic tests and contributed to the redaction of the manuscript. FT, TP, and CB contributed to the interpretation of the results and the redaction of the manuscript. AK and AL followed the patient and contributed to the manuscript. GG contributed to the design of the study, the interpretation of the results and the redaction of the manuscript.

### Conflict of interest statement

The authors declare that the research was conducted in the absence of any commercial or financial relationships that could be construed as a potential conflict of interest.

The reviewer VS and handling Editor declared their shared affiliation.
