# Supplementary material for: Distinct Genomic Features Characterize Two Clades of Corynebacterium diphtheriae: Proposal of Corynebacterium diphtheriae Subsp. diphtheriae Subsp. nov. and Corynebacterium diphtheriae Subsp. lausannense Subsp. nov
Source: Front Microbiol. 2018 Aug 17;9:1743. doi: 10.3389/fmicb.2018.01743 (PMC6108181; doi:10.3389/fmicb.2018.01743)
Supplement: Supplementary file 1 [file Table_1.docx]

Supplementary figures and tables

**Figure S1 – Maximum-likelihood tree based on concatenated MLST sequences.**

Phylogenetic representation based on the alignment of the concatenated 7-loci MLST sequences extracted from all *C. diphtheriae* genomes used in this study (Table S1). The tree was rooted on *C. ulcerans* sequences (ST 327). All lineage-2 isolates (CHUV2995 (ST360), CCUG 5865 (ST106) and CMCNS703 (ST409)) correspond to subspecies *lausannense* and all lineage-1 isolates to subspecies *diphtheriae* (Supplementary file 1). The scale bar represents the number of nucleotide substitutions per site alongside the branches. Nodes supports are based on the Shimodaira-Hasegawa (SH) test.

**Figure S2 – Toxin region**

The genomic region surrounding the toxin gene in NCTC13129 was aligned with CHUV2995 showing the absence of *tox* gene in CHUV2995. Amino acid identity is indicated with the blue bands between the sequences. Interestingly, another prophage, which lacks the *tox* gene, is inserted in CHUV2995 at the same genomic region

**Figure S3 – Heat map of COG counts**

Heatmap showing the COG counts in the dataset. Interestingly, CHUV2995 was enriched in many COG categories. Subspecies *lausannense* were all unusually enriched in categories I (lipid transport and metabolism) and P (inorganic ion transport and metabolism).

**Figure S4 – Number of genes of each COG categories encoded on putative genomic islands**

This graph reflects the number of genes annotated with each COG categories on putative genomic islands of CHUV2995. Putative genomic islands were annotated based on alignment gaps (green regions in Figure 5); regions needed to be larger than 4,500bp and not aligning with more than 80% of the strains. If gaps were located at < 2’000bp from each other, they were merged to omit small regions that were aligned du to repeated or similar sequences and would have wrongly split the mobile genetic element. Interestingly, many COG categories were present in these regions. As expected, the most represented category was COG category X, which includes mobilome-associated genes. For a significant proportion of genes, no COG was assigned.

**Figure S5 – COG counts after subtracting the number of genes annotated on putative genomic islands**

Boxplots representing the number of genes per genome assigned to each COG categories after substracting the counts of the genes annotated on putative genomic islands (Figure S4); we indicated in red, CHUV2995; in green, CMCNS703 and in orange, CCUG 5865 when they are outliers. Interestingly, CHUV2995 remained outlier only in three COG categories (G, I and X), indicating that the enrichment seen in Figure 7 is mainly due to the DNA present on genomic islands. COG categories correspondence: A, Processing and modification; C, Energy production and conversion; D, Cell cycle control, cell division, chromosome partitioning; E, Amino acid transport and metabolism; F, Nucleotide transport and metabolism; G, Carbohydrate transport and metabolism; H, Coenzyme transport and metabolism; I, Lipid transport and metabolism; J, Translation, ribosomal structure and biogenesis; K, Transcription; L, Replication, recombination and repair; M, Cell wall/membrane/envelope biogenesis; N, Cell motility; O, Posttranslational modification, protein turnover, chaperones; P, Inorganic ion transport and metabolism; Q, Secondary metabolites biosynthesis, transport and catabolism; R, General function prediction only; S, Function unknown; T, Signal transduction mechanisms; U, Intracellular trafficking, secretion, and vesicular transport; V, Defense mechanisms; W, Extracellular structures; X, Mobilome: prophages, transposons.

**Figure S6 CRISPR-Cas loci of subspecies *lausannense***

A, Type II-C CRISPR-Cas locus of CHUV2995 is shown. The CRISPR locus had only 2 spacers and is one of the smallest locus described in *C. diphtheriae*; B, Type I-E-a and Type I-E-b loci present in CCUG 5865 with both 19 spacers in the repeat region. The concomitance of type I-E-a and I-E-b was never reported before in *C. diphtheriae*; C Type I-E-a CRISPR-Cas locus of CMCNS703, with only 3 spacers in its region. However, the region might be shorter due to some assembly’s artifacts since repeats are located at the border of a contig.

**Figure S7 Maximum-likelihood tree based on concatenated MLST sequences of subspecies *lausannense***

All concatenated sequences of *C. diphtheriae* 7-loci MLST were downloaded from the PubMLST database ([www.pubmlst.org](http://www.pubmlst.org)) and 78 STs were identified as lineage-2. The Scale bar represents the number of nucleotide substitutions per site alongside the branches. Node supports, based on the Shimodaira-Hasegawa (SH) test, were indicated with an asterisk if they were below 0.8. Labels in blue represent ST types of CHUV2995 (ST360), CCUG 5865 (ST106) and CMCNS703 (ST409).

**Data Sheet 1**

The three sheets correspond to (1) the list of virulence factors assessed in the dataset and displayed in Figure 4, (2) the InterPro domains absent from subspecies *lausannense* and (3) the PFAM domains absent from subspecies *lausannense*.

**Data Sheet 2**

The first sheet contains the detailed annotation of each gene (or coding sequence) found on the genomic islands of CHUV2995 (in regions larger than 4.5Kb that did not align with NUCmer in >80% of the strains) and the second sheet provides the numbers (and statistics) of those genes annotated with each COG category.

**Data Sheet 3**

The first sheet contains all the sequence types (STs) identified in the *C. diphtheriae* strains of the dataset (Table S1) and the second sheet holds the metadata of all subspecies *lausannense* (lineage-2) STs available at the time of submission of the paper.

**Table S1 – List of the strains used for the comparative genomic analysis**

| Species | Strain | BioProject | BioSample | Assembly/Run accession |
| --- | --- | --- | --- | --- |
| *C. diphtheriae* | CHUV2995 | PRJEB24256 | SAMEA104679569 | GCA_900312965 |
| *C. diphtheriae* | TH1526 | PRJNA339927 | SAMN05615426 | GCA_001723445.1 |
| *C. diphtheriae* | TH1141 | PRJNA339923 | SAMN05615420 | GCA_001723455.1 |
| *C. diphtheriae* | TH510 | PRJNA339932 | SAMN05615463 | GCA_001723465.1 |
| *C. diphtheriae* | TH1337 | PRJNA342907 | SAMN05771005 | GCA_001742085.1 |
| *C. diphtheriae* | TH2031 | PRJNA342909 | SAMN05771014 | GCA_001742095.1 |
| *C. diphtheriae* | c123 | PRJNA345527 | SAMN05877646 | GCA_001832925.1 |
| *C. diphtheriae* | c325 | PRJNA345527 | SAMN05877647 | GCA_001832935.1 |
| *C. diphtheriae* | c122 | PRJNA345527 | SAMN05877645 | GCA_001832945.1 |
| *C. diphtheriae* | c110 | PRJNA345527 | SAMN05877644 | GCA_001832975.1 |
| *C. diphtheriae* | c488 | PRJNA345527 | SAMN05877648 | GCA_001833005.1 |
| *C. diphtheriae* | c517 | PRJNA345527 | SAMN05877649 | GCA_001833025.1 |
| *C. diphtheriae* | c20 | PRJNA345527 | SAMN05877642 | GCA_001833035.1 |
| *C. diphtheriae* | NCTC 13129 | PRJNA224116 | SAMEA1705951 | GCF_000195815.1 |
| *C. diphtheriae* | 31A | PRJNA224116 | SAMN02603066 | GCF_000241875.1 |
| *C. diphtheriae* | 241 | PRJNA224116 | SAMN02603070 | GCF_000241895.1 |
| *C. diphtheriae* | INCA 402 | PRJNA224116 | SAMN02603075 | GCF_000241915.1 |
| *C. diphtheriae* | BH8 | PRJNA224116 | SAMN02603077 | GCF_000241935.1 |
| *C. diphtheriae* | HC03 | PRJNA224116 | SAMN02603073 | GCF_000242775.1 |
| *C. diphtheriae* | HC02 | PRJNA224116 | SAMN02603072 | GCF_000255155.1 |
| *C. diphtheriae* | C7 (beta) | PRJNA224116 | SAMN02603067 | GCF_000255175.1 |
| *C. diphtheriae* | HC04 | PRJNA224116 | SAMN02603074 | GCF_000255195.1 |
| *C. diphtheriae* | CDCE 8392 | PRJNA224116 | SAMN02603069 | GCF_000255215.1 |
| *C. diphtheriae* | HC01 | PRJNA224116 | SAMN02603071 | GCF_000255235.1 |
| *C. diphtheriae* | VA01 | PRJNA224116 | SAMN02603076 | GCF_000255255.1 |
| *C. diphtheriae* | PW8 | PRJNA224116 | SAMN02603068 | GCF_000255275.1 |
| *C. diphtheriae* | NC03529 | PRJNA224116 | SAMN02471955 | GCF_000257885.1 |
| *C. diphtheriae* | NCTC 5011 | PRJNA224116 | SAMN02471953 | GCF_000263415.1 |
| *C. diphtheriae* | DSM 43988 | PRJNA224116 | SAMN02471956 | GCF_000455785.1 |
| *C. diphtheriae* | str. Aberdeen | PRJNA224116 | SAMN02471951 | GCF_000455805.1 |
| *C. diphtheriae* | HC07 | PRJNA224116 | SAMN03104762 | GCF_000953975.1 |
| *C. diphtheriae* | 17801 | PRJNA224116 | SAMN03396925 | GCF_000968865.1 |
| *C. diphtheriae* | ISS 4060 | PRJNA224116 | SAMN02837548 | GCF_001026805.1 |
| *C. diphtheriae* | ISS 3319 | PRJNA224116 | SAMN02837547 | GCF_001026825.1 |
| *C. diphtheriae* | ISS 4746 | PRJNA224116 | SAMN02837549 | GCF_001026845.1 |
| *C. diphtheriae* | ISS 4749 | PRJNA224116 | SAMN02837550 | GCF_001188005.1 |
| *C. diphtheriae* | NCTC 11397 | PRJNA224116 | SAMEA2517360 | GCF_001457455.1 |
| *C. diphtheriae* | Cd_1/usb1 | PRJEB14914 | SAMEA104349899 | ERR2173761 |
| *C. diphtheriae* | Cd_2/usb2 | PRJEB14914 | SAMEA104349900 | ERR2173762 |
| *C. diphtheriae* | Cd_3/usb3 | PRJEB14914 | SAMEA104349901 | ERR2173763 |
| *C. diphtheriae* | Cd_4/usb4 | PRJEB14914 | SAMEA104349902 | ERR2173764 |
| *C. diphtheriae* | Cd_6/usb6 | PRJEB14914 | SAMEA104349903 | ERR2173765 |
| *C. diphtheriae* | Cd_7/usb7 | PRJEB14914 | SAMEA104361022 | ERR2183212 |
| *C. diphtheriae* | Cd_8/usb8 | PRJEB14914 | SAMEA104349905 | ERR2173766 |
| *C. diphtheriae* | Cd_9/usb9 | PRJEB14914 | SAMEA104349906 | ERR2173767 |
| *C. diphtheriae* | Cd_10/usb10 | PRJEB14914 | SAMEA104345680 | ERR2167138 |
| *C. diphtheriae* | Cd_11/usb11 | PRJEB14914 | SAMEA104349992 | ERR2173855 |
| *C. diphtheriae* | Cd_15/usb15 | PRJEB14914 | SAMEA104349993 | ERR2173856 |
| *C. diphtheriae* | Cd_16/usb16 | PRJEB14914 | SAMEA104349994 | ERR2173857 |
| *C. diphtheriae* | Cd_17/usb17 | PRJEB14914 | SAMEA104349995 | ERR2173858 |
| *C. diphtheriae* | Cd_18/usb18 | PRJEB14914 | SAMEA104349913 | ERR2173769 |
| *C. diphtheriae* | Cd_19/usb19 | PRJEB14914 | SAMEA104349914 | ERR2173770 |
| *C. diphtheriae* | Cd_20/usb20 | PRJEB14914 | SAMEA104349915 | ERR2173771 |
| *C. diphtheriae* | Cd_B/usbB | PRJEB14914 | SAMEA104349917 | ERR2173773 |
| *C. diphtheriae* | Cd_C/usbC | PRJEB14914 | SAMEA104361023 | ERR2183213 |
| *C. diphtheriae* | CCUG 5865 | PRJNA313138 | SAMN04515814 | GCA_002200975.1 |
| *C. diphtheriae* | CMCNS703 | PRJNA357968 | SAMN06163065 | GCA_001935945.1 |
| *C. ulcerans* | BR-AD22 | PRJNA61801 | SAMN02603079 | GCA_000215645.1 |
| *C. pseudotuberculosis* | C231 | PRJNA40875 | SAMN02604117 | GCA_000144675.1 |
| *C. jeikeium* | K411 | PRJNA13967 | SAMEA3283089 | GCA_000006605.1 |

Species, strains, bioprojects, biosamples and accession numbers of the strains included in the comparative genome analysis.

**Table S2 – API Corynebacterium results**

| Strain | NIT | PYZ | PyrA | PAL | βGUR | βGAL | αGLU | βNAG | ESC | URE | GEL |
| --- | --- | --- | --- | --- | --- | --- | --- | --- | --- | --- | --- |
| CHUV2995 | - | - | - | - | - | - | - | - | - | - | - |
| CHUV1863 | + | - | - | - | - | - | + | - | - | - | - |
| CHUV2453 | + | - | - | - | - | - | + | - | - | - | - |

| Strain | 0 | GLU | RIB | XYL | MAN | MAL | LAC | SAC | GLYG | CAT |
| --- | --- | --- | --- | --- | --- | --- | --- | --- | --- | --- |
| CHUV2995 | - | + | + | - | - | + | - | - | - | + |
| CHUV1863 | - | + | + | - | - | + | - | - | - | + |
| CHUV2453 | - | + | + | - | - | + | - | - | - | + |

Strain CHUV2995 was surprisingly negative for the alpha-glucosidase activity as well as for the nitrate reductase activity. Two *C. diphtheriae* strains isolated in the Lausanne University Hospital were used as controls. Both CHUV1863 and CHUV2453 were identified as *C. diphtheriae* biovar mitis or belfanti.
