# Supplementary figures and images for: Distinct Genomic Features Characterize Two Clades of Corynebacterium diphtheriae: Proposal of Corynebacterium diphtheriae Subsp. diphtheriae Subsp. nov. and Corynebacterium diphtheriae Subsp. lausannense Subsp. nov
Source: Front Microbiol. 2018 Aug 17;9:1743. doi: 10.3389/fmicb.2018.01743 (PMC6108181; doi:10.3389/fmicb.2018.01743)

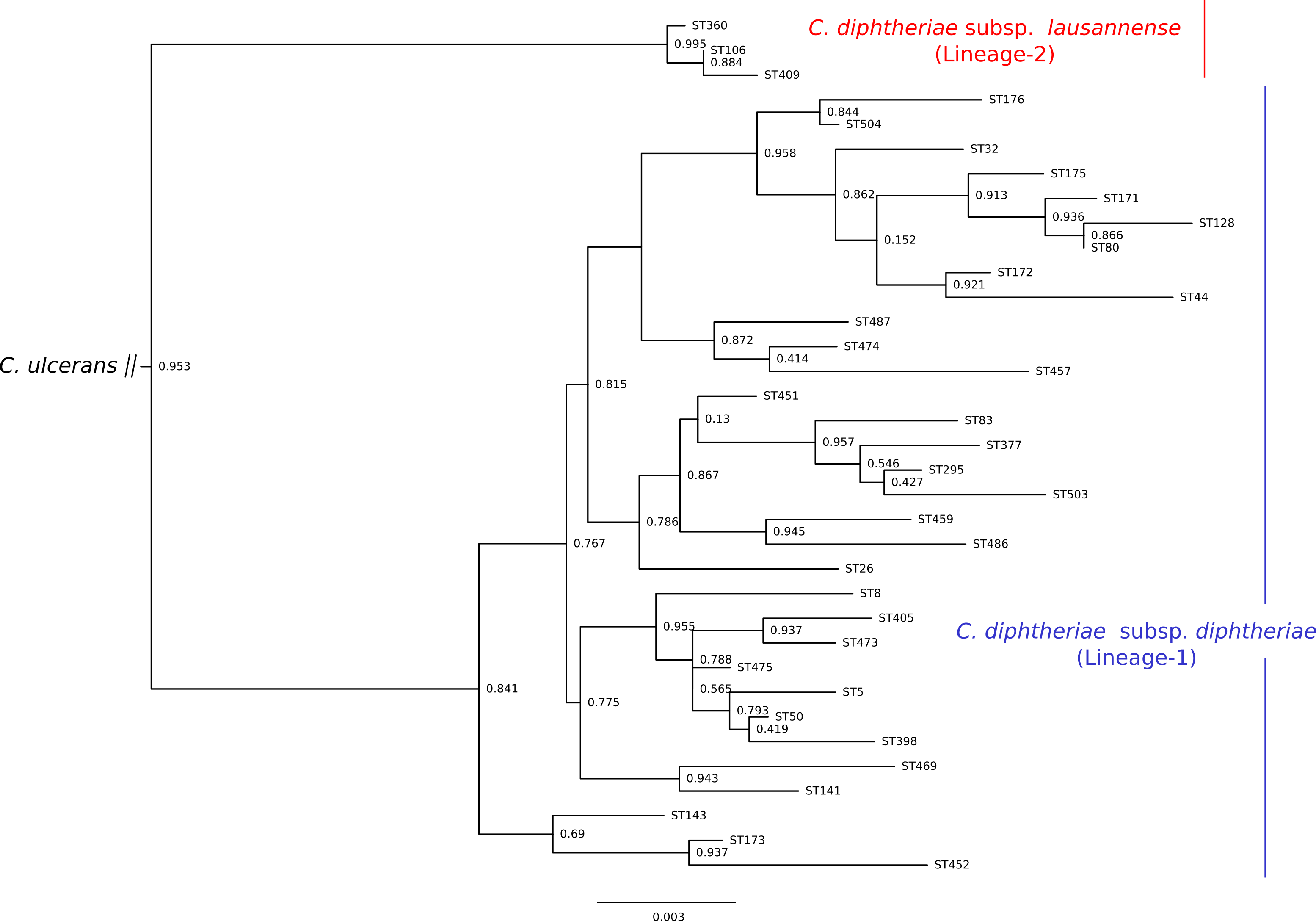

Supplement: Supplementary file 5 [file Image_1.TIFF]

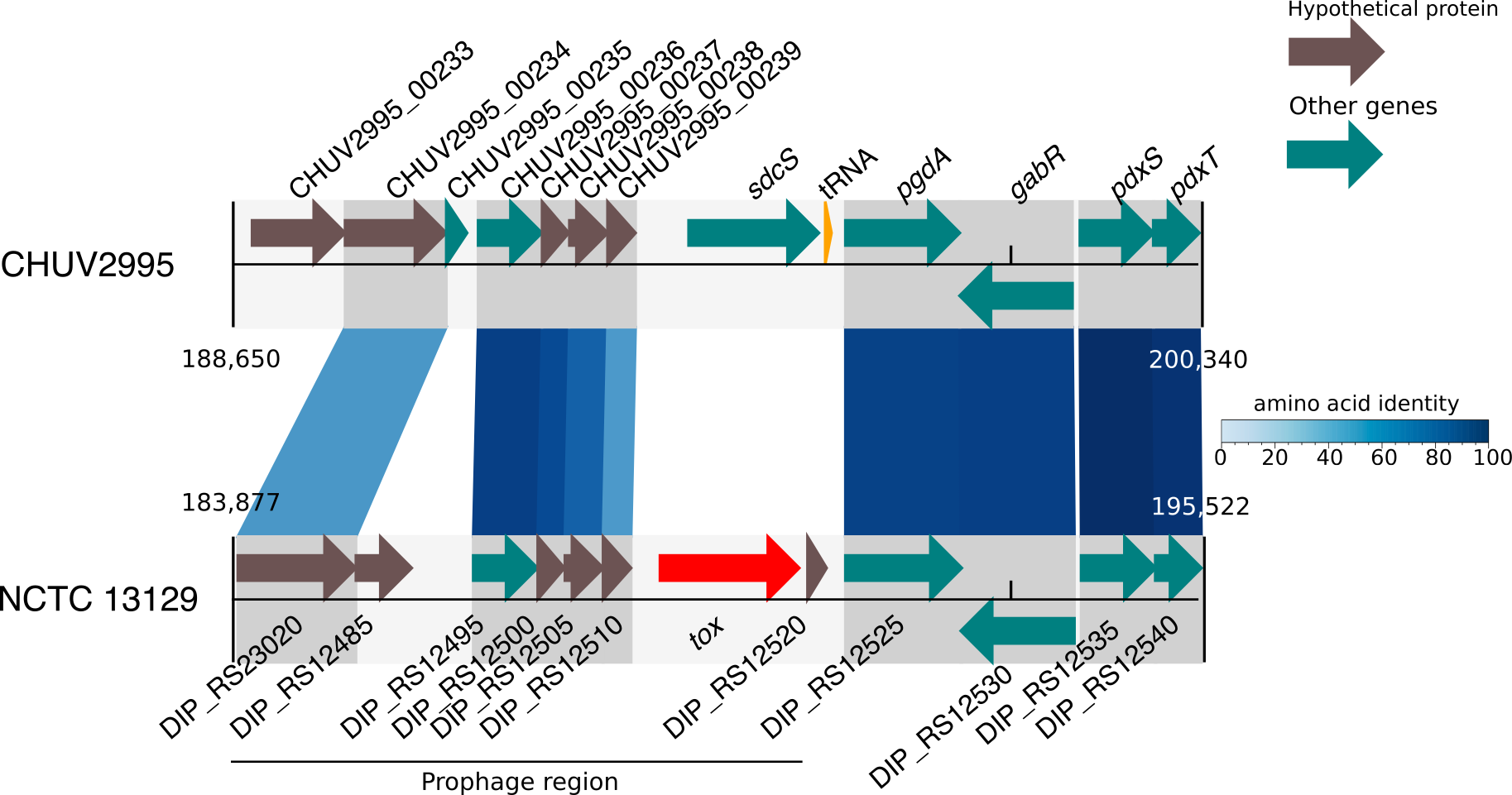

Supplement: Supplementary file 6 [file Image_2.TIFF]

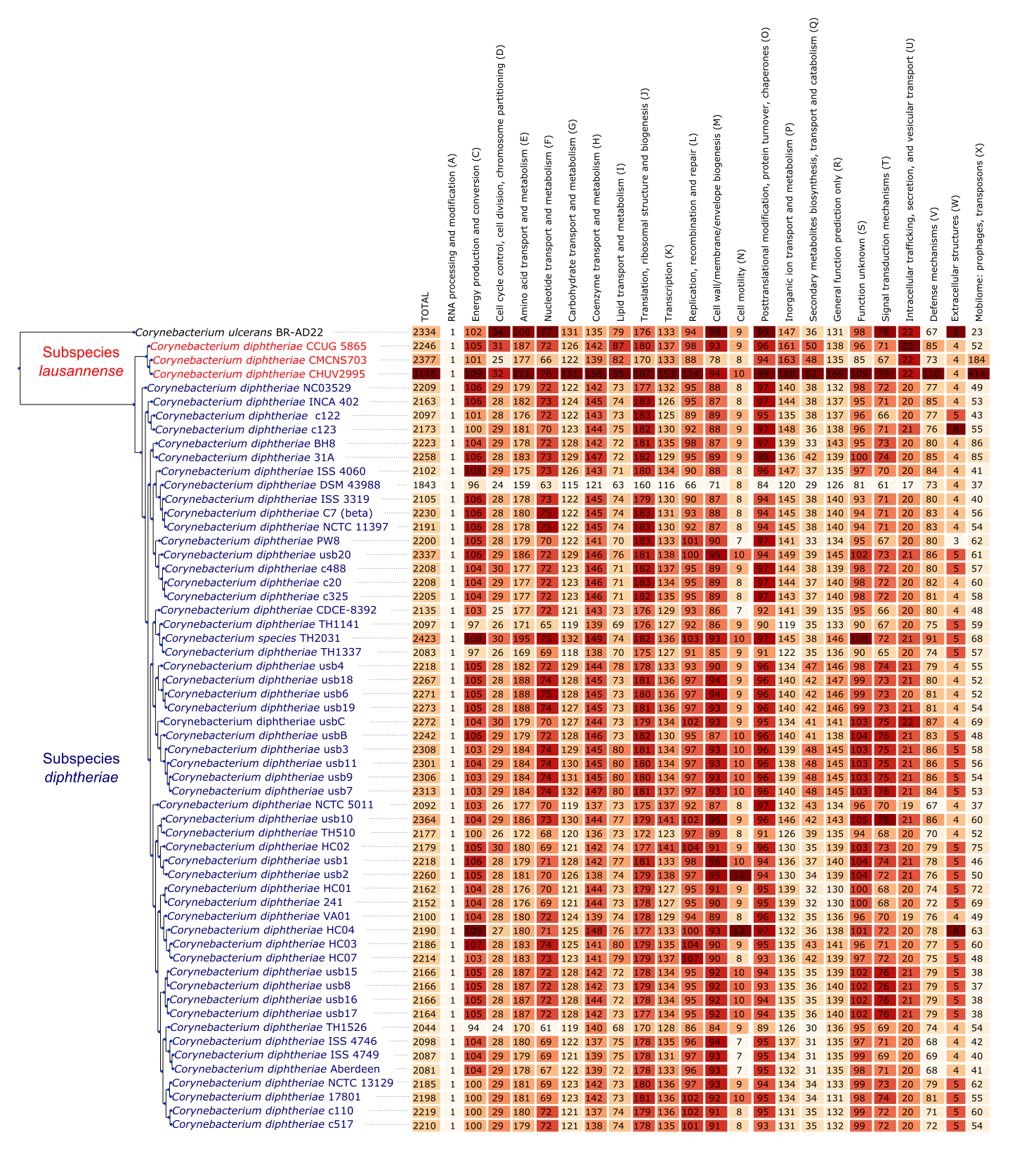

Supplement: Supplementary file 7 [file Image_3.TIFF]

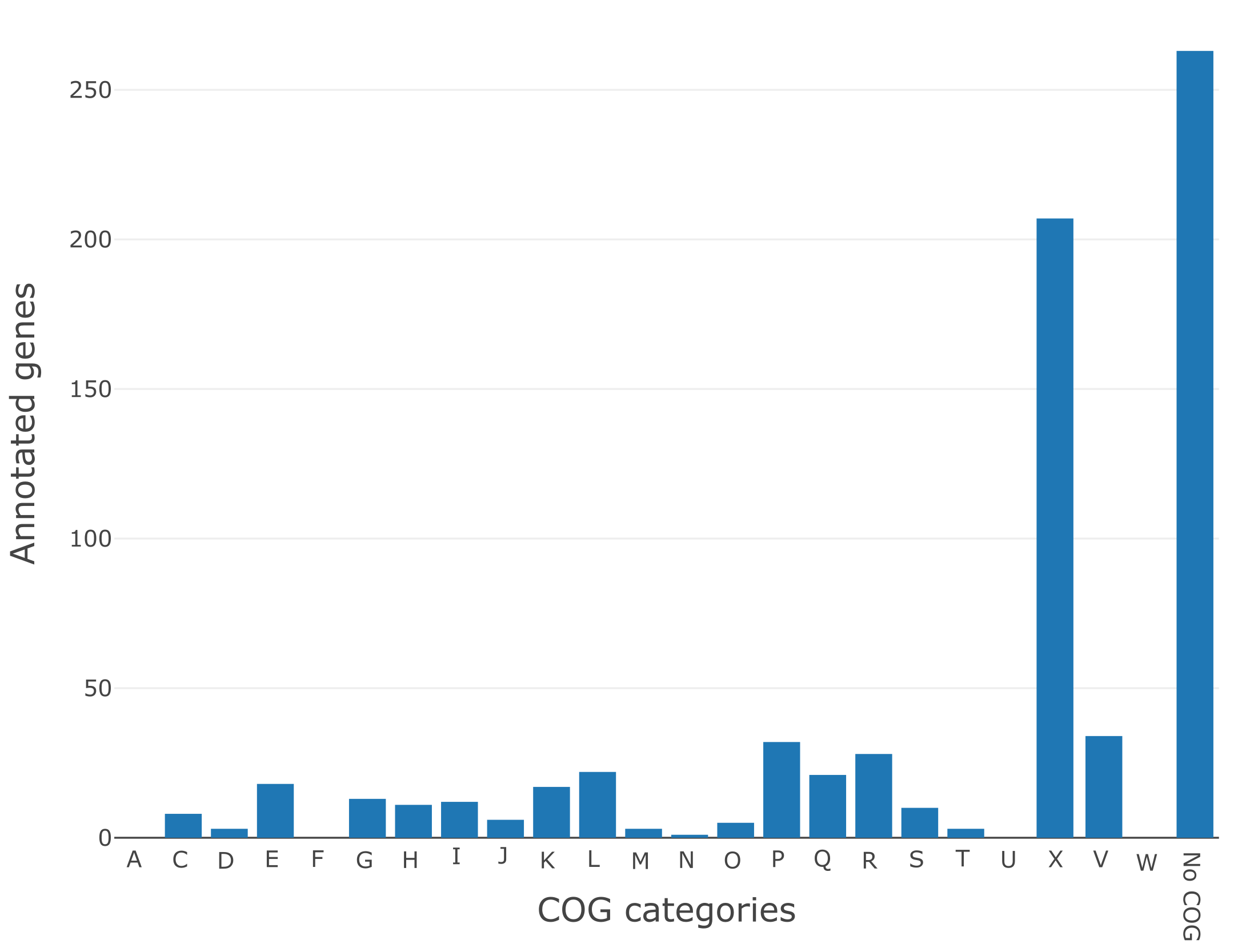

Supplement: Supplementary file 8 [file Image_4.TIFF]

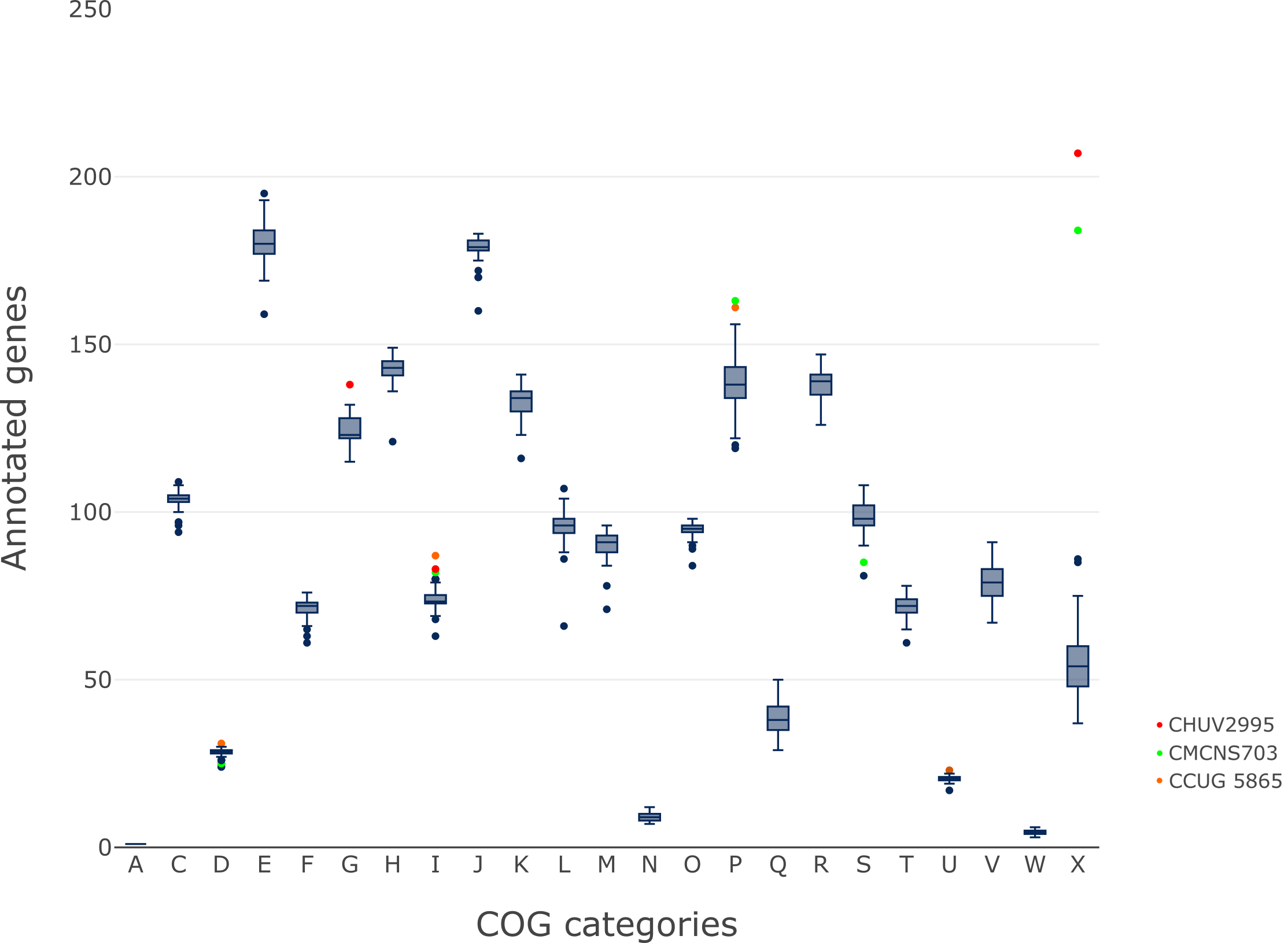

Supplement: Supplementary file 9 [file Image_5.TIFF]

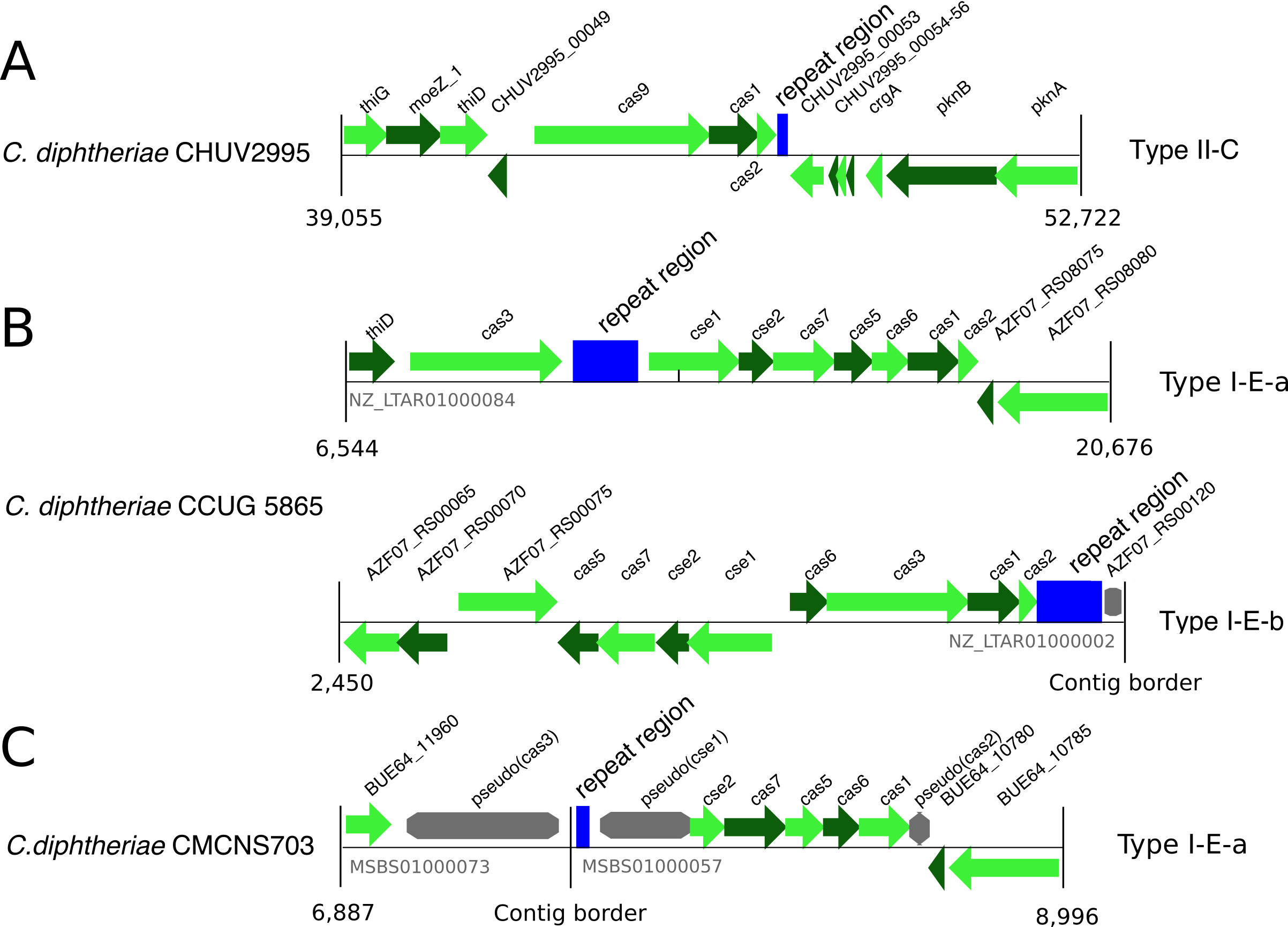

Supplement: Supplementary file 10 [file Image_6.TIFF]

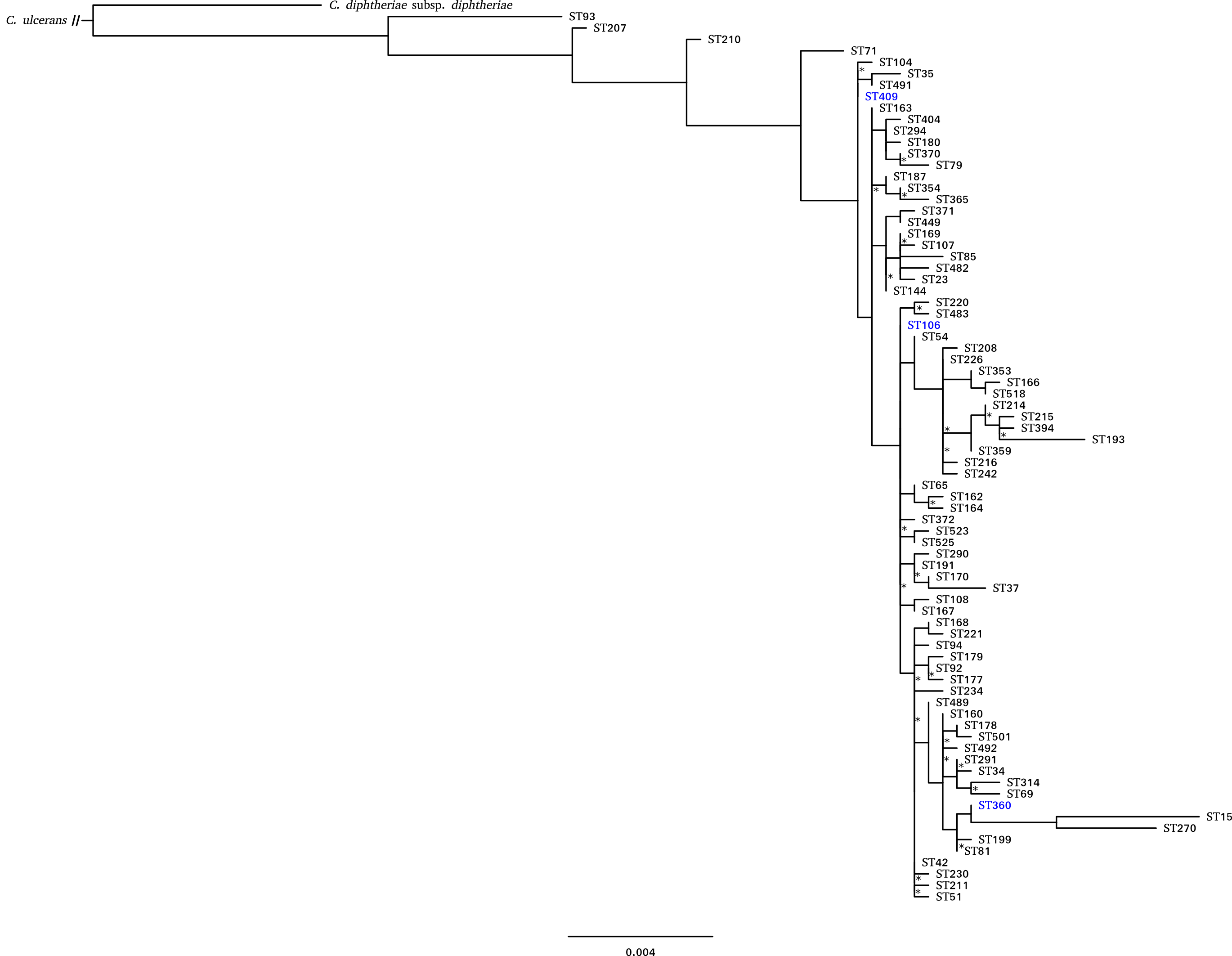

Supplement: Supplementary file 11 [file Image_7.TIFF]
